# Signaling pathways and targeted therapies in lung squamous cell carcinoma: mechanisms and clinical trials

**DOI:** 10.1038/s41392-022-01200-x

**Published:** 2022-10-05

**Authors:** Zhenyi Niu, Runsen Jin, Yan Zhang, Hecheng Li

**Affiliations:** 1 Department of Thoracic Surgery, Ruijin Hospital, Shanghai Jiao Tong University School of Medicine, 197 Ruijin 2nd Road, Shanghai, China; 2 Department of Immunology and Microbiology, Shanghai Jiao Tong University School of Medicine, 280 South Chongqing Road, Shanghai, China

**Keywords:** Molecular medicine, Lung cancer

## Abstract

Lung cancer is the leading cause of cancer-related death across the world. Unlike lung adenocarcinoma, patients with lung squamous cell carcinoma (LSCC) have not benefitted from targeted therapies. Although immunotherapy has significantly improved cancer patients’ outcomes, the relatively low response rate and severe adverse events hinder the clinical application of this promising treatment in LSCC. Therefore, it is of vital importance to have a better understanding of the mechanisms underlying the pathogenesis of LSCC as well as the inner connection among different signaling pathways, which will surely provide opportunities for more effective therapeutic interventions for LSCC. In this review, new insights were given about classical signaling pathways which have been proved in other cancer types but not in LSCC, including PI3K signaling pathway, VEGF/VEGFR signaling, and CDK4/6 pathway. Other signaling pathways which may have therapeutic potentials in LSCC were also discussed, including the FGFR1 pathway, EGFR pathway, and KEAP1/NRF2 pathway. Next, chromosome 3q, which harbors two key squamous differentiation markers *SOX2* and *TP63* is discussed as well as its related potential therapeutic targets. We also provided some progress of LSCC in epigenetic therapies and immune checkpoints blockade (ICB) therapies. Subsequently, we outlined some combination strategies of ICB therapies and other targeted therapies. Finally, prospects and challenges were given related to the exploration and application of novel therapeutic strategies for LSCC.

## Introduction

Lung cancer is the leading cause of cancer-related death across the world.^1^ In 2020, lung cancer remained the leading cause of cancer deaths, with an estimated 1.8 million deaths.^2^ Lung cancer was traditionally classified into two primary groups, small versus non-small-cell type. Approximately 85% of lung cancers are non-small-cell lung cancer (NSCLC). Lung adenocarcinoma (LUAD) and lung squamous cell carcinoma (LSCC) are the most common subtypes, with the latter accounting for approximately 30% of all NSCLC.^3,4^ The 5-year survival rate for NSCLC is 26%, and only 24% of lung cancers are diagnosed at a localized stage, for which the 5-year survival rate is 60%.^5^ The mortality rate of lung cancer has declined largely in the past few decades, with the pace accelerating in recent years due to major advances in treatment for NSCLC.^5,6^ Over the past 20 years, the treatment of NSCLC has evolved from the empirical use of cytotoxic therapy to effective and better-tolerated regimens by targeting specific molecular subtypes in both LUAD^7^ and LSCC.^4^ This improvement is largely attributed to the accumulation of molecular knowledge and the discovery of targeted molecular abnormalities. Previously, the treatment options for these two subtypes of NSCLC were historically similar.^8^ In 2004, two studies found out that a subgroup of NSCLC patients with specific mutations in the epidermal growth factor receptor (EGFR) gene were correlated with clinical responses to the tyrosine-kinase inhibitor (TKI) gefitinib.^9,10^ These findings ushered in a widening divide in the management of these two diseases.

While the steady identification of new oncogenic alterations and development of new targeted drugs continue to prolong the progression-free survival (PFS) and the overall survival (OS) in LUAD patients, it was of note that the use of targeted therapy in LSCC patients has been associated with negative outcomes in early-phase studies.^11–19^ In fact, both the mutated genes and the recurrent somatic copy numbers vary widely between these two diseases.^4,20^ The common driver mutations in LUAD, like EGFR and KRAS, are rarely mutated in LSCC.^21,22^ The biomarker-driven therapies for LSCC evaluated in the Lung Cancer Master Protocol (Lung-MAP; S1400) failed to show an improvement of therapeutic effect of currently available targeted therapy with an overall response rate of only 7%.^15,17,19,23^ This has changed with the advent of immune-checkpoint blockade (ICB) therapy, which has the potential to transcend the histological boundaries and, most importantly, achieve sustained remission in patients.^24^ Immunotherapies, such as anti-programmed cell death 1 (PD-1)/programmed cell death ligand 1 (PD-L1) therapy, lead to durable responses and manageable adverse effects.^25^ Currently, pembrolizumab and atezolizumab were both approved by the US Food and Drug Administration (FDA) as the first-line monotherapy in patients with advanced LSCC with PD-L1 levels of 50% or more.^26–28^ In addition, pembrolizumab plus chemotherapy is also recommended as a first-line therapy in patients with metastatic LSCC.^29^ Other ICB therapies, including nivolumab,^30,31^ ipilimumab,^30,31^ cemiplimab,^32^ sintilimab,^33^ tislelizumab,^34^ camrelizumab,^35^ and sugemalimab,^36^ have also significantly improved the outcomes of patients with advanced LSCC. However, a large proportion of LSCC patients still do not respond to current immunotherapy. Identification of biomarkers for immunotherapy and exploration of more effective therapeutics remains to be an unmet need for LSCC patients.

In this review, we will discuss the current knowledge of potentially targetable molecular alterations in LSCC as well as providing some updated information about ongoing or finished clinical trials which may change or have changed the therapeutic landscapes of LSCC.

## Genomic landscapes of LSCC and therapeutic targets

**Fig. 1:**
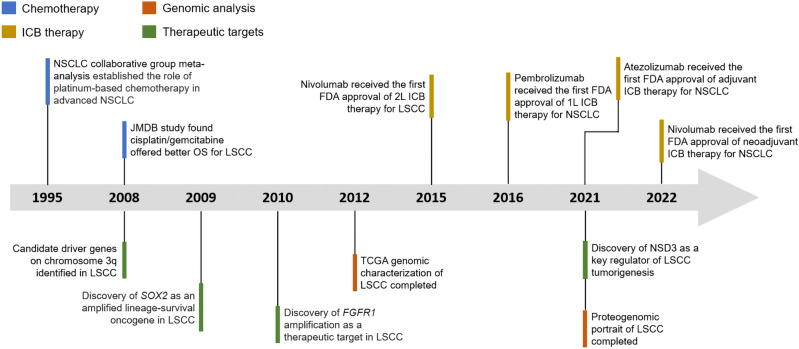
Timeline illustrating the evolving treatment landscapes and research history of LSCC. Timeline highlights some of the pivotal clinical studies and discoveries that might change or have changed the management of LSCC. 1 L first line, 2 L second line, ICB therapy immune-checkpoint blockade therapy, OS overall survival, TCGA The Cancer Genome Atlas

LSCC is strongly associated with smoking and is characterized by a high overall mutation rate of 8.1 mutations per megabase and marked genomic complexity.^4^ A number of significantly mutated genes have been identified in LSCC: *TP53*, *CDKN2A*, *PTEN*, *PIK3CA*, *KEAP1*, *MLL2*, *HLA-A*, *NFE2L2*, *NOTCH1*, and *RB1*, all of which demonstrated robust evidence of gene expression.^4,37^ Almost all LSCC displays somatic mutation of *TP53*. The amplification between chromosome 3q26 and 3q28 represents a hallmark for LSCC, which harbors key squamous differentiation markers *SOX2* and *TP63.*^4,37–40^ In addition, other significantly amplified regions include 8p11 (*FGFR1*, *WHSC1L1*), 7p11 (*EGFR*), 11q13 (*CCDN1*) and 4q12 (*KDR*, *KIT*, *PDGFRA*).^37,41^ Although *EGFR* mutations were found in 7% of the LSCC cases, there were no activating mutations of exon 19 deletions or Leu858Arg substitution.^4^ These mutated genes cause frequent alterations in the following signaling pathways: *CDKN2A/RB1*, *NFE2L2/KEAP1/CUL3*, *PI3K/AKT*, and *SOX2/TP63/NOTCH1* pathways, some of which play an important role in cell-cycle control, response to oxidative stress, apoptotic signaling, and squamous cell differentiation.^4,37^ These signaling pathways are interconnected with each other. It has been shown that both mutated genes and recurrent somatic copy-number alterations are largely distinct in LSCC and LUAD.^20^ In fact, an analysis of 12 cancer types revealed the convergence of squamous-like subtype, suggesting similarities in genomic- and pathway-based determinants in four different tumor types: LSCC, head and neck squamous cell carcinoma (HNSCC), some bladder urothelial carcinoma and a very few LUAD.^42,43^ Besides frequent alterations in different signaling pathways caused by mutated genes and amplification in the chromosome 3q region, aberrant protein lysine methylation modification also influences other cellular pathways in LSCC. Recently, NSD3—a histone dimethyltransferase encoded by an *FGFR1*-neighboring gene—was identified as a major mutational driver in LSCC.^44^ Other histone modifiers, including SETD8,^45^ LSD1^38,46,47^ and EZH2,^38,48,49^ have also been identified to be involved in LSCC tumorigenesis and the inhibition of these targets could produce potent antitumoral effects. In the last 10 years, the great benefits that ICB therapy have demonstrated in several clinical trials heralded a new era in the management of LSCC. For resectable NSCLC, compared to traditional cytotoxic chemotherapy alone, the addition of ICB therapy both in the neoadjuvant and adjuvant settings has significantly improved the outcomes of patients.^50,51^ We briefly summarized some of the pivotal clinical studies and discoveries that might change or have changed the management of LSCC in the last thirty years (Fig. 1).

## Targeting signaling pathways in LSCC

**Fig. 2:**
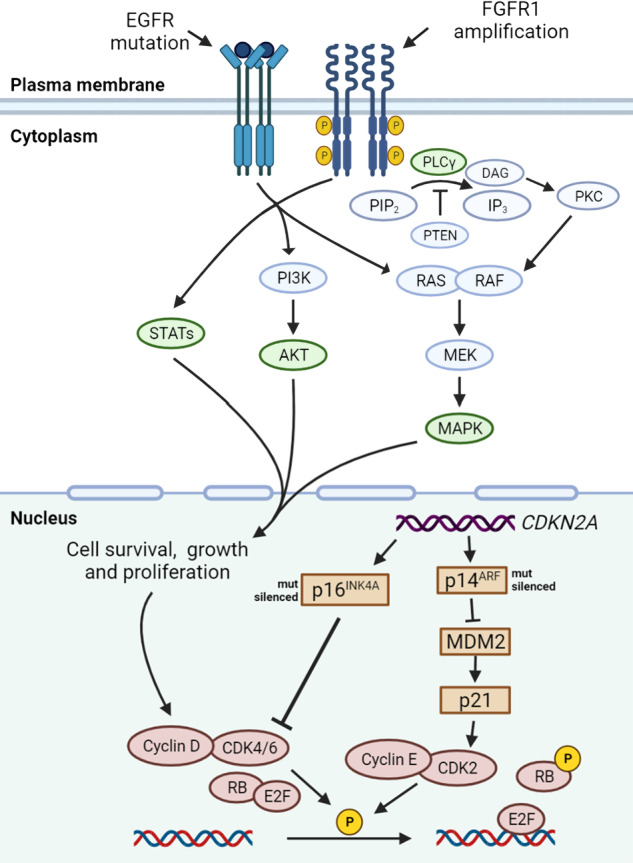
Mutated driver genes and downstream signal pathways in LSCC. *FGFR1* amplification is observed in 20% of LSCC patients. *FGFR1* amplifications can lead to overexpression of wild-type FGFR1 proteins on the cell membrane, resulting in increased sensitivity to FGF and the promotion of tumor growth via increased activation of four key downstream signaling pathways: PLCγ, PI3K-AKT, RAS-MAPK, and STAT (green). Although mutations in *EGFR* gene are relatively rare in LSCC, in certain cases of LSCC these mutations are responsible for constitutive ligand-independent receptor activation and downstream signaling, promoting cell survival and proliferation. Mitogenic signaling, including activation of upstream PI3K and MAPK signaling, could drive cyclin D upregulation, which leads to CDK4/6 activation. The phosphorylation of RB by cyclin D-CDK4/6 complexes and cyclin E-CDK2 complexes releases E2F transcriptional factors to activate genes required for G1-S transition. The *CDKN2A* gene encodes p16^INK4A^ and p14^ARF^, which regulate cell cycle by inhibiting CDK4/6 and CDK2, respectively. In LSCC, the inactivated *CDKN2A* caused by genomic alterations may upregulate this pathway. DAG diacylglycerol, E2F E2 family, FRS2 FGFR substrate 2, GRB2 growth factor receptor-bound 2, IP3 inositol triphosphate, MDM2 murine double minute 2, P phosphorylation, PIP2 phosphatidylinositol-4,5-biphosphate, PKC protein kinase C, PLCγ phospholipase Cγ, PTEN phosphatase and tensin homolog, STAT signal transducer and activator of transcription

In the past few decades, multiple mutated genes in LSCC have been discovered. As we mentioned in the last section, these mutated genes cause alterations in several signaling pathways. In this section, we would discuss pathways proved in other cancer types but not in LSCC, including PI3K signaling pathway, VEGF/VEGFR signaling, and CDK4/6 pathway. Other signaling pathways in LSCC which may be potentially targeted were also discussed, including FGFR1 pathway, EGFR pathway, and KEAP1/NRF2 pathway. It is of note that these signaling pathways are also closely connected with each other (Fig. 2).

### The classical PI3K pathway

The PI3K signaling pathway is one of the most frequently altered signaling pathways in human cancer,^52–55^ which can be activated by various growth factors/ligands specific to different RTKs, including members of the EGFR family, and the insulin and insulin-like growth factor 1 (IGF-1) receptor, FGF, etc.^56^ LSCC has high rates of alterations in the PI3K pathway, and alterations were found in 68% of LSCC samples.^57^ Activation of PI3K signaling pathway mediated through molecular aberrations is instrumental in promoting tumor development as well as resistance to antitumor therapies.^55,58^ PI3K belongs to a family of lipid kinases, which are classified into three different classes based on structural features and lipid substrate preferences.^59^ Class I PI3K is frequently implicated in cancer. Class IA consists of the PIK3CA, PIK3CB, and PIK3CD genes, and encode for the catalytic subunit of p110α, p110β, and p110δ, respectively. Class IB includes PIK3CG coding for p110γ.^54^ Whereas p110α and p110β show broad tissue distribution, p110δ and p110γ are highly enriched in all leukocyte subtypes.^60^

A common mechanism of PI3K activation in cancers is through the presence of mutations in the PIK3CA gene.^61^ The reported incidence of PIK3CA alterations in LSCC varies between 8 and 20% and the two main mutation types are canonical PIK3CA mutations and PIK3CA amplification.^62–64^ The canonical PIK3CA mutations affect two different domains of p110α, the kinase domain and the helical domain.^65^ These two types of mutations can activate the downstream signaling through two distinctive mechanisms. The kinase domain mutations can change the dynamics of the membrane-binding surface and affect the PIP_2_ substrate.^66^ The helical domain mutations (e.g., E542K, E545K) abrogate the inhibitory interactions between p110α and the N-terminal SH2 domain of the p85 regulatory subunit, leading to constitutive activity that mimics pTyr stimulation.^66,67^

Mutations in other genes may also lead to abnormal activation of the PI3K pathway. Phosphatase and tensin homolog (*PTEN*) is a 9-exon tumor-suppressor gene located on chromosome 10q23. This gene encodes for a 403-amino acid protein with dual lipid and protein phosphatase utility which contains four functional domains: an N-terminal PI(4,5)P2-binding/phosphatase, domain, a C2 domain, a carboxyl-terminal tail domain (C-tail), and a PDZ-binding domain (PDZ-BD).^68^ This protein classically dampens the PI3K/AKT/mTOR growth-promoting signaling cascade by directly dephosphorylating phosphatidylinositol-3,4,5-trisphosphate (PIP_3_) and converting it back to the phosphatidylinositol-4,5-bisphosphate (PIP_2_) inactive state.^69,70^ Accordingly, PTEN dysfunction causes dysregulation of this and other pathways, resulting in tumorigenesis and cancer progression.^71–73^ *PTEN* is mutated in 7–10% of LSCC^62,63,74^ and these somatic mutations tend to be distributed across its 9 exons. Some tumor-associated missense mutations may lead to complete loss or severe impairment of the phosphatase activity of the encoded enzyme.^75–77^ Many tumor-derived PTEN mutants retain partial or complete catalytic function, suggesting that alternative mechanisms can lead to the inactivation of PTEN.^78^ In addition to its own genetic alterations, *PTEN* gene expression is also regulated at epigenetic,^79^ transcriptional,^80–82^ post-transcriptional^83–86^, and post-translational^87–92^ levels as well as by protein–protein interactions.^93,94^

Partial loss of PTEN function can have dramatic effects on tumorigenesis and cancer progression,^71,72^ reflecting the fact that PTEN is a haploinsufficient tumor suppressor. PTEN function is not often completely lost in cancer, providing an opportunity to reactivate its function as a mode of cancer treatment. It has been reported that a tumor-suppressive metabolic state is induced in transgenic mouse lines with the systemic elevation of PTEN.^95^ In 2019, Lee et al.^96^ reported a way to reactivate PTEN by inhibiting the MYC-WWP1 inhibitory pathway. In the study, they identified the HECT-type E3 ubiquitin ligase WWP1 as a physical PTEN interactor, the amplification, and overexpression of which may lead to pleiotropic inactivation of PTEN. A natural and potent WWP1 inhibitor, indole-3-carbinol (I3C), was also found to effectively suppress tumorigenesis driven by the PI3K-AKT pathway. Therefore, both genetic and pharmacological targeting of the MYC-WWP1 axis may be a viable approach for cancer patients driven by impaired PTEN function.

Although extensive research has been conducted on the PI3K pathway component genes as potential molecular therapeutic targets in human cancers in the past two decades, clinical success to date has been limited to the approval of the PI3K inhibitors for hematological malignancies and breast cancer.^34,97–101^ Even among the four current FDA-approved PI3K inhibitors for the treatment of hematological malignancies, some of the indications have been withdrawn from the marketing authorization application.^102–105^ Although some of the decisions were made, according to the manufacturing companies, based on business needs, this will certainly have implications for the future of PI3K inhibitors. In NSCLC, the early-phase clinical trials of PI3K inhibitors and dual PI3K/mTOR inhibitors have yielded negative results.^16–18^ The modest therapeutic efficacy of PI3K inhibitors may be attributed to various reasons, including insufficient target inhibition, intrinsic and acquired drug resistance, and tolerability.^106^ Unlike other oncogenes, such as *EGFR* in LUAD, the correlation between specific PI3K pathway mutations and drug sensitivity is not absolute.^107^ This makes patient selection more complicated based on PI3K pathway mutation status. Another issue that cannot be overlooked about PI3K inhibitors is their on-target, off-tumor toxicity, particularly hyperglycemia and hyperinsulinemia which are observed as major dose-limiting toxicities.^98,108,109^ Despite the benefits of PFS shown in several randomized clinical trials, the increased toxicities of PI3K inhibitors have raised concerns about the potential detriments of OS in the PI3K inhibitor arm.^34,105,110–113^ In blood cancers, future approvals of PI3K inhibitors by FDA should be supported by randomized data.^114^

It has been suggested that alternative dosing regimens which offer intermittent pathway inhibition can increase the therapeutic window without compromising therapeutic efficacy.^115–117^ Another solution is to develop selective compounds that are more selective for mutant PI3K than wild-type PI3K. In the future, more durable therapeutic responses could be achieved by a more tailored PI3K-based therapies with a better understanding of the role of PI3K in cancer and surrounding environments.

### Cell cycle in LSCC: the CDK4/6 pathway

The *CDKN2A* locus, located on human chromosome 9p21, is one of the most common genetic losses in human cancer.^118,119^ TCGA profiling of 178 LSCC samples revealed that *CDKN2A* is inactivated in 72% cases of LSCC.^4^ The *CDKN2A* locus encodes two alternatively spliced proteins, p16INK4a (p16) and p14ARF (p14), which function as cell-cycle inhibitors. These two tumor-suppressor proteins function in distinct anticancer pathways: p16 regulates retinoblastoma (RB), and p14 regulates p53. RB is a tumor-suppressor protein which controls cell cycle by preventing entry into the DNA synthesis (S) phase of the cell-division cycle.^120^ The p16 protein directly inhibits the activities of the cyclin D-dependent kinases, cyclin-dependent kinase (CDK) 4 and CDK6, thus maintaining RB in its dephosphorylated, anti-proliferative state, and leading to cell growth arrest.^121^ The tumor-suppressor protein p53 plays a pivotal role in regulating cell growth following exposure of cells to various stress stimuli.^122^ The p14 protein associates directly with murine double minute 2 (MDM2), a negative regulator of p53, preventing the export and degradation of p53.^123–126^

At present, the therapeutic focus has been on leveraging CDK4/6 inhibition to activate RB and limit tumor cell proliferation to delay disease progression.^127,128^ Interesting to note, the pan-caner analysis of the CDK4/6 pathway showed that *CDKN2A* loss and *RB1* loss were mutually exclusive in most cancers that lose these genes at a significant level (>5%).^129^ The proteogenomic portrait of LSCC revealed that loss of one of these two key CDK4/6 pathway inhibitors is a universal feature of LSCC.^38^ However, CDK4/6 inhibitors have shown minimal efficacy in LSCC clinical trials.^19,130–132^ Phospho-RB levels have been shown to be correlated with response to CDK4/6 inhibitors in various LSCC cell lines.^38^ The heterogeneity of RB expression and phosphorylation may provide a reasonable explanation for the diverse responses toward CDK4/6 inhibitors. The screening of tumors based on the downstream functional assessment (i.e., RB expression and phosphorylation) may identify tumors that are sensitive to CDK4/6 inhibitors.

### VEGF-VEGFR signaling in LSCC

Vascular endothelial growth factor (VEGF, here referred to as VEGF-A) is a member of a protein family that also includes VEGF-B, VEGF-C, VEGF-D, VEGF-E (a virally encoded protein), and placental growth factor (PIGF, also known as PGF).^133^ VEGF-B has multifaceted and context-dependent functions that safeguard the balance between blood vessel growth and degeneration to ensure normal blood vessel density and integrity.^134^ VEGF-C and VEGF-D are mainly implicated in lymphangiogenesis.^135^ As VEGF-A plays a dominant role in regulating angiogenesis and disease, it is referred to as VEGF in this review. Alternative exon splicing causes multiple isoforms of VEGF which are characterized by their differential ability to bind heparin.^136^ VEGF binds to both VEGF receptor 1 (R1) and VEGFR2 while VEGFR2 is the main receptor for VEGF.^137,138^ VEGF isoforms can also interact with the neuropilin co-receptors (NRP1 and NRP2).^139,140^ During tumorigenesis, angiogenesis plays a key role in maintaining the expansion in tumor. Most human tumors overexpressed VEGF mRNA, and its expression correlates with invasiveness, increased vascular density, metastasis, tumor recurrence and poor prognosis.^141^ Accordingly, several strategies that target this VEGF-VEGFR signaling has been devised.^142,143^

Neutralizing monoclonal antibodies (mAbs) against VEGF have shown great effect in preclinical studies^144^ and were the first type of antiangiogenic drugs that entered the market. In 2004, bevacizumab was approved by the FDA for the treatment of metastatic colorectal cancer based on the results of AVF2107 clinical trial.^145^ However, as the benefits of bevacizumab extended to other malignancies, including non-squamous NSCLC, renal cell carcinoma, ovarian cancer, and cervical cancer,^142^ LSCC is not one of them, as clinical trials have shown that bevacizumab increases the risk of life-threatening pulmonary hemorrhages in squamous cell carcinomas.^146,147^ Another antiangiogenic agent, ramucirumab, a human IgG1 monoclonal antibody targeting the extracellular domain of VEGFR2, is currently the only antiangiogenic agent that is approved by FDA for the treatment of LSCC. Based on the results of phase III REVEL clinical trial,^147^ ramucirumab plus docetaxel is recommended as a subsequent therapy option for metastatic NSCLC following disease progression on or after platinum-based chemotherapy.^148^

### FGFR1 pathway

Fibroblast growth factor receptor 1 (FGFR1) belongs to the FGFR family of receptor tyrosine kinases (RTKs), which consists of four members: FGFR1 to FGFR4. All these four members share a canonical RTK architecture, consisting of a large ligand-binding extracellular domain that comprises three immunoglobulin-like domains (D1-3) followed by a single transmembrane helix and an intracellular domain containing the catalytically active “split” tyrosine-kinase domain.^149,150^ There is also a fifth related receptor, FGFR5 (also known as FGFRL1), which lacks the cytoplasmic tyrosine-kinase domain.^151^ The native ligand of FGFRs is fibroblast growth factors (FGFs), which can be divided into two categories: hormone-like FGFs (i.e., FGF19, 21, and 23) and canonical FGFs (i.e., FGF1-10, 16–18, and 20).^150,152^ The intracellular signaling of the FGFR pathway is primarily mediated mainly through four key downstream pathways: RAS-RAF-MAPK pathway, PI3K-AKT, signal transducer and activator of transcription (STAT), and phospholipase Cγ (PLCγ)^153–155^ (Fig. 1). Dysregulation of FGFR signaling promotes the proliferation,^156^ survival^157^ and development of drug resistance^158^ in tumor cells, as well as the development of angiogenesis^159^ and immune evasion in the tumor microenvironment (TME).^160^ These findings make FGFR pursued as a potential therapeutic target and support the development of FGFR-targeting anticancer agents.

*FGFR1* amplifications are the predominant type of *FGFR* mutation, occurring in nearly 20% of LSCC patients.^160,161^ Although the studies in preclinical models have suggested that FGFR inhibitors may be a viable therapeutic option in this cohort of patients,^161,162^ a number of FGFR-specific small molecular inhibitors tested in phase I and phase II trials have shown modest effects with overall response rates of 8–15%.^11–15^ The results from these trials suggest that *FGFR1* amplification is not a reliable predictor of response to FGFR1 inhibitors and that FGFR1 mutations have a more complex impact in NSCLC than EGFR-mutated or ALK-rearranged NSCLC.^163^ A previous study has found that elevated *FGFR1* mRNA and/or protein expression was often independent of *FGFR1* amplification.^164^ Future studies are needed to clarify the role of FGFR1 signaling in the pathogenesis of LSCC.

### EGFR pathway

EGFR belongs to the HER/*erb*B family of RTKs, which includes HER1 (EGFR/*erb*B1), HER2 (*neu*, *erb*B2), HER3 (*erb*B3), and HER4 (*erb*B4). All members display similar structures: an extracellular, cysteine-rich ligand-binding region, a single alpha-helix membrane-spanning region and a cytoplasmic tyrosine-kinase-containing domain.^165^ The intracellular signaling of EGFR pathway is mediated mainly through the RAS/MAPK pathway, the PI3K pathway, and the STAT pathway.^166,167^ Downstream EGFR signaling ultimately leads to increased proliferation,^168^ angiogenesis,^169^ metastasis,^170^ and decreased apoptosis.^171^ Alterations in EGFR signaling pathways result in constitutive activation of its kinase activity and the inhibition of tumor apoptosis, leading to a poor clinical outcome.^172,173^ All these findings make EGFR pursued as therapeutic targets and support the development of EGFR-targeting anticancer agents.^174^

The reported rate of *EGFR* mutation in LSCC patients is 4.2–7%,^4,175,176^ which is much lower compared with LUAD patients. In previous prospective phase III clinical trials assessing the efficacy of first-line EGFR-TKIs in the treatment of NSCLC, only 27 cases of LSCC patients with *EGFR* mutation were identified in six clinical trials, which were further randomized into two groups.^177–182^ This limited number of LSCC cases makes it hard to assess the benefits of EGFR-TKIs for *EGFR-*mutated LSCC in prospective studies. Subgroup analysis in the BR.21 and SATURN clinical trials showed that erlotinib was effective in unselected LSCC patients.^183,184^ A meta-analysis also confirmed that EGFR-TKIs demonstrated an improved OS and PFS compared to placebo in unselected patients with advanced LSCC.^185^ Based on previous retrospective matched-pair studies,^186,187^ EGFR-TKIs were less effective in *EGFR*-mutant LSCC than in LUAD but still had clinical benefits for LSCC patients. Another retrospective study found that in Chinese female *EGFR*-mutant LSCC, EGFR-TKIs conferred longer PFS and OS than chemotherapy, but the survival was similar with patients without *EGFR* mutations.^188^ In conclusion, for *EGFR*-mutant LSCC, EGFR-TKIs can improve the outcomes of these patients compared with chemotherapy, but its efficacy is not as robust as that of EGFR-TKIs for *EGFR*-mutant LUAD.

Notably, EGFR protein was significantly upregulated in the squamous cancers but not in LUAD,^38^ although many activating *EGFR* mutations occurred in LUAD. This *EGFR* amplified LSCC cohort did not show elevated EGFR pathway activity,^189^ but displayed a high correlation with mRNA abundance of the five EGFR ligands. This is consistent with the results in HNSCC,^190^ which indicates a squamous cell carcinoma feature that EGFR ligand abundance drives the activity of EGFR pathways. It suggests that EGFR ligand abundance, rather than *EGFR* amplification, might be a better predictor for EGFR inhibitor response in this population of LSCC patients.

### KEAP1/NRF2 pathway

**Fig. 3:**
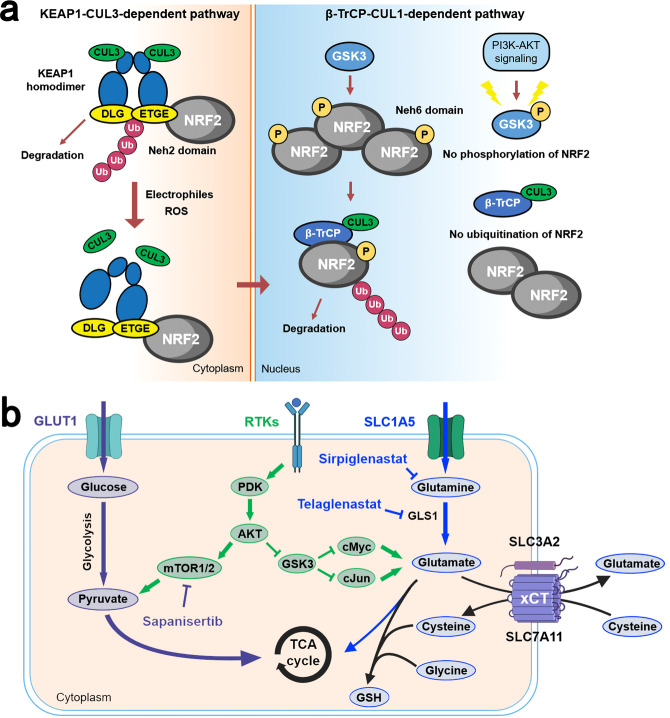
Physiologic activation and regulation of NRF2 and metabolic reprogramming by NRF2 in LSCC cells. **a** In unstressed conditions, KEAP1 forms a ubiquitin E3 ligase complex with CULLIN3 (CUL3) and binds with NFR2 via the DLG and ETGE motifs in the Neh2 domain of NRF2 in the cytoplasm. NRF2 is then polyubiquitinated and degraded through the proteasome system after its synthesis. When cells are exposed to electrophiles or ROS, KEAP1 is modified and the KEAP1-CUL3 ubiquitin E3 ligase activity declines, which stabilizes NRF2. Stabilized and accumulated NRF2 translocates to the nucleus and functions as a transcriptional factor. NRF2 is also regulated through a KEAP1-independent mechanism in which GSK3 plays an important role. NRF2 is phosphorylated by GSK3 and then recognized by β-TrCP. By contrast, the Neh6 domain of NRF2 serves as the degron exploited in this β-TrCP-CUL1-dependent degradation of NRF2. Following its ubiquitination by the β-TrCP-CUL1 E3 ubiquitin ligase complex, NRF2 is degraded by the proteasome. **b** LSCC cells displayed a dual reliance on glucose and glutamine metabolism. Activation of NRF2 increases the synthesis of GSH from intracellular glutamate, cysteine, and glycine. GLS1 catalyzes the transformation of glutamine to glutamate. Cystine is imported by the x_c_^–^ antiporter system (xCT). Serine and glycine are synthesized via NRF2-dependent processes. Under chronic mTOR inhibition which suppresses glycolysis, LSCC cells could upregulate glutaminolysis through the GSK3 signaling pathway which developed acquired resistance to mTOR inhibition. β-TrCP β-transducin repeat-containing protein genes, GLS1 glutaminase 1, GSH glutathione, PDK phosphoinositide-dependent kinase, ROS reactive oxygen species, TCA cycle tricarboxylic acid cycle

The Kelch-like ECH-associated protein 1 (KEAP1, encoded by *KEAP1*)/nuclear factor erythroid 2-related factor 2 (NRF2, encoded by *NFE2L2*) pathway plays a physiologic protective role against environmental insults.^191^ This KEAP1-NRF2 system operates as a typical two-component system: KEAP1 as a sensor for insults, NRF2 as an effector for the coordinated activation of cytoprotective genes (Fig. 3a). The NRF2 signaling is primarily regulated by KEAP1 in response to reactive oxygen species (ROS) and electrophiles,^192,193^ but also by the PI3K signaling pathway,^194^ with glycogen synthase kinase 3 (GSK3) acting as a key mediator^195^ (Fig. 3a). In addition to crosstalk with the PI3K-AKT signaling pathway, the KEAP1-NRF2 system also interacts with the autophagy pathway through the adaptor p62.^196^ Hyperactivation of NRF2 plays a critical role in promoting both tumorigenesis and resistance to multiple therapies,^197–201^ resulting from the mutually exclusive loss-of-function (LOF) mutations in *KEAP1* or gain-of-function (GOF) mutations in *NRF2*. Furthermore, there is also evidence that NRF2 has its tumor-preventive role during initiation,^202^ suggesting that the dual stage-specific pro- and anti-tumorigenic effects of NRF2 are context-dependent.

Alterations in the KEAP1/NRF2 pathway are significantly enriched in lung and upper airway cancers. In LSCC, mutations in *NRF2* are more prevalent while *KEAP1* mutations are more common in LUAD.^4,203–205^ KEAP1/NRF2 pathway mutations were found in approximately one third of patients with LSCC. As we mentioned earlier, NRF2 has a dual function during carcinogenesis: preventing cancer initiation and promoting tumor progression. This is supported by the fact that *NRF2*-null mice exhibit an increased level of lung metastasis compared with wild-type mice after cancer cell incubation,^206^ while activated NRF2 in tumor cells is associated with poor prognosis and more aggressive disease.^199,207^ This contribution of NRF2 to the malignant phenotype of tumor cells is independent of its antioxidant activities and is associated with its key role in metabolic reprogramming of cancer cells. LSCC cells displayed a dual reliance on glucose and glutamine metabolism.^208^ The metabolic enzymes that are involved in this pentose phosphate pathway and glutamine metabolism were found to be directly activated through the antioxidant response element (ARE).^209^ NRF2 also regulates the intracellular abundance of these amino acids. *SLC7A11* is a NRF2 target gene encoding SLC7A11, which dimerizes with SLC3A2 to form the x_c_^−^ antiporter system (xCT).^210^ xCT functions as a concentration-dependent antiporter, which exports glutamate while importing cystine, the dimerized form of cysteine. Three amino acids were required in the synthesis of glutathione: glutamate, cysteine, and glycine, and xCT can keep intracellular stores of cysteine for glutathione synthesis.^209,210^ This NRF2-mediated depletion of intracellular glutamate stores either through export (xCT) or consumption (glutathione synthesis) makes highly metabolic LSCC cells dependent on extracellular glutamine import, which is transformed to glutamate by GLS1^211^ (Fig. 3b). This vulnerability of tumor cells can be targeted through the inhibition of the activity of GLS1. Another possible therapeutic target is targeting the PI3K-AKT pathway. PI3K-AKT signaling is a major proliferative signal that inactivates GSK3 by phosphorylation, which increases NRF2 in a KEAP1-independent way (Fig. 3a). The GSK3 pathway is also a key regulator of adaptive glutamine metabolism.^208^

Based on this preclinical evidence, therapeutic interventions targeting the KEAP/NRF2 signaling have been tested in clinical trials. Three treatment approaches were used to target this signaling, including glutaminase inhibition, glutamine antagonism, and PI3K-AKT signaling inhibition. However, the phase II KEAPSAKE study which evaluated telaglenastat, a glutaminase inhibitor, in advanced non-squamous NSCLC with *KEAP1/NRF2* mutations was terminated due to lack of clinical benefits among patients treated with telaglenasta in the interim analysis.^212^ For PI3K-AKT signaling inhibition, an mTOR kinase inhibitor sapanisertib was evaluated in a phase II clinical trial for advanced or recurrent LSCC with KEAP1/NRF2 mutations (NCT02417701). The preliminary results showed an objective response rate (ORR) of 25% (3/12) in the *NFEL2* mutant squamous cohort and 16.7% (1/6) in the *KEAP1* mutant squamous cohort.^213^ The relatively low response rate in these two cohorts may be attributed to the circumvention of mTOR inhibition by upregulation of glutamine metabolism through GSK3 signaling axis in LSCC cells (Fig. 3b).^208^ Glutamine blockade seems to be a promising strategy, which has been shown to induce divergent metabolic programs between cancer cells and effector T cells, acting as a “metabolic checkpoint” for tumor immunotherapy.^214^ Currently, sirpiglenastat, a broad glutamine antagonist, is being evaluated in combination with atezolizumab for patients with advanced solid tumors in phase I/IIa clinical trial (NCT04471415).^215^

## Therapeutic targets on chromosome 3Q in LSCC

*SOX2* and *TP63* are both regarded as key squamous differentiation markers located on chromosome 3q.^38,39^ The suppression of *SOX2* gene in LSCC cell lines had the highest anti-proliferative effects among the immediate genes on chromosome 3q26.33, including *PIK3CA, TP63*, *DCUND1.*^40^ It was also found that LSCC cell lines amplified for *SOX2* and *TP63* were highly dependent on them, supporting oncogene addiction.^38^

SOX2 belongs to the sex-determining region Y (SRY)-related high-mobility-group (HMG) box family characterized by a DNA-binding HMG domain, which shares at least 46% of sequence homology across all sox proteins.^216^ As a transcription factor, SOX2 recognizes and binds to the promoter of various target genes with complex, important, and pleiotropic impacts in multiple tissues in development and maintaining homeostasis.^217,218^ Dysregulation of *SOX2* expression is an important factor contributing to cancer pathogenesis,^219,220^ associated with several tumor features, including cancer stemness,^221–224^ cell proliferation and migration,^225–227^ apoptosis,^228–230^ and drug resistance.^231–234^

*TP63* encodes p63, a transcription factor that belongs to the tumor-suppressor p53 superfamily of transcription factors, including two additional paralogs, p53 and p73.^235,236^ The three members of the p53 family share very significant homology both at the genomic and at the protein level. Each contains a transactivation domain (TAD), a DNA-binding domain (DBD) and an oligomerization domain (OD).^237^ A common feature of all p53 family members is that they can be expressed in many different isoforms.^236,238^ For p63, three different splice variants are characterized by their differences in their C-termini: a full-length α form; a β form that is truncated after exon XII; and a γ form that lacks exons XII-XIV and uses an additional exon XV.^238^ Each of these isoforms can be further divided into TA forms and ∆N forms, based on the presence of the TAD or not, which is decided by whether transcription of the precursor mRNA starts from exon I (TA forms) or from exon III′ (∆N forms). Physiologically, p63 plays a critical role in sustaining epithelial development and morphogenesis in the regulation of epithelial proliferation.^239,240^ However, in the control of tumorigenesis, TA-isoforms and ∆N-isoforms shared antagonistic properties. TAp63 functions as tumor suppressors to halt tumorigenesis^241^ while ∆Np63 is more associated with promoting tumorigenesis.^242,243^

### SOX2

Given that *SOX2* is amplified in various types of cancer and involved in tumorigenesis via complicated signaling pathways and protein–protein interactions, targeting SOX2 is a promising strategy for anticancer therapy.^40^ Previously, as a transcription factor, SOX2 was deemed undruggable because of its absence of active sites or allosteric regulatory pockets to be targeted by small molecule inhibitors (SMIs).^244^ Therefore, studies targeting SOX2 in anticancer therapy has been focusing on the upstream and downstream signaling of SOX2. Recently, Liu et al.^245^ reported the development of a platform using the technique of proteolysis-targeting chimeras (PROTACs), which is able to selectively degrade the transcription factors of interest. This generalizable platform may help target SOX2 as an effective anticancer therapy.

### ∆Np63

The main isoform of p63 expressed in adult squamous tissues is ∆Np63.^246^ For squamous cell carcinomas (SCCs), ∆Np63 acts as a proto-oncogenic transcription factor and the master regulator of SCC formation.^247–250^ The oncogenic potential of ∆Np63 is related to its direct competition with p53, TAp63, and TAp73 on the same p53 responsive elements and the consequent inhibition.^251,252^ High levels of endogenous ΔNp63 protein abundance are essential to induce and maintain SCC tumors.^247,253^ Acute gene ablation of ΔNp63 in an autochthonous SCC model could induce rapid tumor regression.^253^ Besides, ΔNp63 is also found to regulate chemoresistance in SCCs by controlling the expression of DNA repair genes.^254,255^ Collectively, these findings implicate that ΔNp63 is a promising therapeutic target in LSCC. As a transcription factor, ΔNp63 was considered undruggable, as with most transcription factors which lack suitable domains for the binding of SMIs.^244^ The development of a generalizable platform by Liu et al.^245^ based on the technique of PROTACs, which is able to selectively degrade the transcription factors of interest, may provide new strategies to target ∆Np63. However, it is of note that ∆Np63 is associated with the regulation of a massive subset of different genes and cellular processes, which makes complete blocking of ∆Np63 almost impossible.

### USP28

Ubiquitin-specific peptidase 28 (USP28) belongs to the largest deubiquitinating enzyme family, which removes ubiquitin from the ubiquitin conjugates.^256^ ΔNp63 is tightly regulated at the protein level by the ubiquitin-proteasome system, which can be targeted by multiple E3 ligases.^257^ USP28 is highly abundant in SCCs and correlates with poor prognosis.^258^ In SCCs, USP28 could stabilize ΔNp63 and maintain elevated ΔNp63 levels by counteracting its proteasome-mediated degradation.^258^ The researchers further confirmed that the pharmacologic inhibition of USP28 showed a selective anti-proliferative response of SCC cells.^258^ In addition to its tumor-suppressive function, inhibition of USP28 in ΔNp63 expressing SCC could sensitize SCC cells to cisplatin treatment by toning down the DNA damage response pathways.^259^ Taken together, these data show that USP28-ΔNp63 axis is required in the maintenance of SCC identity and control of SCC marker gene.

USP28 stabilizes ΔNp63 independently of FBXW7,^258^ which is a component of SCF (complex of SKP1, CUL1, and F-box protein)-type ubiquitin ligases.^260^ FBXW7 is a tumor suppressor that binds to key regulators of cell division and growth, including cyclin E, MYC, JUN, and Notch, most of which are proto-oncogenes that are closely related to the pathogenesis of human cancers.^261^ Recurrent mutations in the FBXW7 tumor-suppressor gene have been reported in LSCC.^20,262^ FBXW7 and USP28 are closely related in that USP28 could lead to FBXW7 substrate accumulation (either via destabilization of FBXW7 or via stabilization of both FBXW7 and its substrates).^263,264^ Therefore, targeting USP28 to destabilize the substrates of FBXW7 represents a promising strategy to inhibit the function of MYC and other oncogenic regulators.

Inhibition of USP28 is particularly effective in mouse LSCC models, resulting in dramatic tumor regression.^258,265^ The USP28 inhibitor used by Prieto-Garcia et al.^258^ was AZ1, a dual USP25/USP28 inhibitor, while the USP28 inhibitor FT206 used by Ruiz et al.,^265^ preferentially inhibits USP28 compared to USP25. Despite evidence that USP25 is an oncoprotein,^266^ its oncogenic function in LSCC is still enigmatic. There is currently no specific inhibitor of USP28 mainly due to the highly similar catalytic structure of USP25 and USP28. In the future, with the help of novel drug development technologies, USP28 inhibitors may become a promising therapeutic option for LSCC, but further clinical trials are still needed.

### Survivin

Survivin (also known as BIRC5) has been a well-known cancer therapeutic target since its discovery over 20 years ago.^267^ Because of its essential role in cell mitosis and inhibition of apoptosis,^268–270^ as well as its variable expression levels in cancer and normal cells,^271^ survivin appears be a ideal candidate for anticancer therapy. However, no survivin-specifc drugs have yet reached the clinic. SMIs and inhibitory peptides targeting survivin for NSCLC have been explored in clinical trials but have shown modest or no improvement.^272–274^

Recently, Satpathy et al.^38^ identified ΔNp63-low LSCC which showed no elevation at RNA or protein levels. Accordingly, they also discovered a substantial number of LSCC cell lines with low ΔNp63 expression which were significantly more vulnerable to the survivin inhibitor YM-155. These findings may provide new strategies for selecting LSCC patients based on the TP63 status, which may have better response to survivin inhibition.

### TNIK

TRAF2-/NCK-interacting kinase (TNIK) is a member of germinal center kinase (GCK) family, which was found previously involved in the promotion of colorectal cancer, triple-negative breast cancer (TNBC), prostate cancer, and chronic myelogenous leukemia.^275–279^ Furthermore, this protein kinase was identified as a potential genetic dependency in tumors with distal amplification of the 3q chromosome.^280^ *TNIK* gene is amplified in ~50% of LSCC cases.^63^ In LSCC cell lines with high *TNIK* expression, depleting TNIK can significantly reduce their growth.^281^ In addition, TNIK inhibition also showed antitumor activity and increased apoptosis in established LSCC patient-derived xenografts. These findings suggest TNIK as a potential therapeutic target for LSCC patients with *TNIK* gene copy-number gains.

## Epigenetic therapeutic targets in LSCC

Epigenetics is defined as the DNA sequence-independent inheritance of phenotype or gene expression.^282^ There are four major mechanisms of epigenetic regulation: DNA methylation, histone post-translational modifications, chromatin structure regulation, and noncoding RNA regulation.^282^ In cancer cells, the epigenetic features are commonly dysregulated. High rate of alterations in many epigenetic regulator genes was observed in cancer genome-sequencing studies.^283^ This plethora of genetic lesions in epigenetic regulators has attracted much attention as possible targets for the development of epidrugs. Most epidrugs that has been approved by FDA are for the treatment of hematopoietic malignancies.^284^ Tazemetostat, a EZH2 inhibitor, was approved by FDA for advanced epithelioid sarcoma, making it the first epidrug to treat solid tumors.^285^ In this chapter, we will discuss some potential epigenetic therapeutic targets in LSCC.

### NSD3: the neighboring gene of FGFR1

**Fig. 4:**
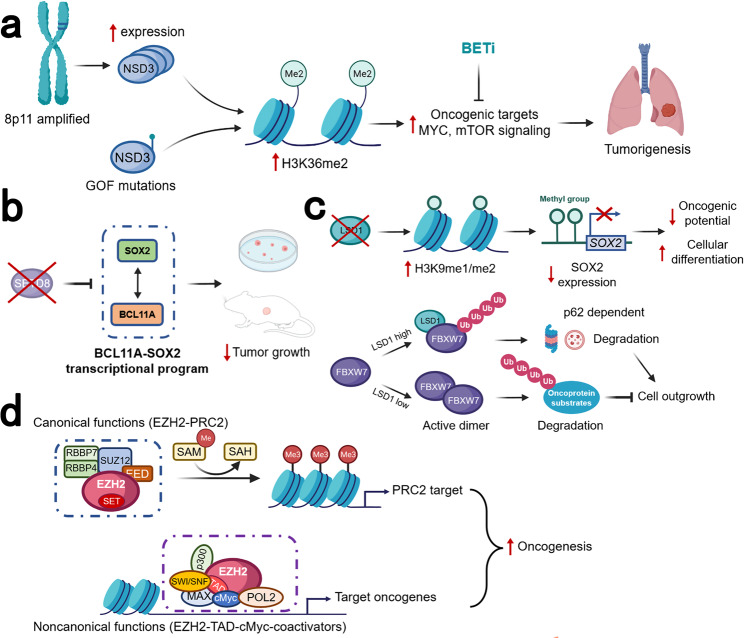
Schematic diagram of the different roles for epigenetic therapeutic targets in LSCC. **a** A recent study suggested that *NSD3*, the neighboring gene of *FGFR1*, rather than *FGFR1*, was the critical driver oncogene within this recurrent focal amplicon of 8p11-12 genomic region. The amplification of *NSD3* leads to increased NSD3 expression, thus increasing the synthesis of H3K36me2. Less common than the amplification of 8p11-12 and NSD3 expression, the GOF variant NSD3 was also present in LSCC. These two works together to increase H3K36me2, stimulating transcription of oncogenic targets, including mTOR pathways and MYC-associated pathways. This process rendered the tumor NSD3-addicted, which could be inhibited by BETi. **b** *SOX2* and *BCL11A* are both identified as LSCC oncogenes. The BCL11A-SOX2 transcriptional program is crucial for the maintenance of a squamous phenotype. SETD8 is a monomethyltransferase, whose gene is regulated by SOX2 and BCL11A. The inhibition of SETD8 selectively limits LSCC tumor growth. **c** LSD1 could promote tumorigenesis in two different ways. The first way is demethylase-dependent. In SOX2-expressing tumor cells, LSD1 inhibition will induce increased H3K9me1/me2. The repressive H3K9 methylations act on the *SOX2* gene, leading to SOX2 downregulation, reduced oncogenic potential, and increased cellular differentiation. The second way is demethylase-independent. In cells with a low level of LSD1, FBXW7 forms a dimer, which promotes ubiquitylation for proteasomal degradation of oncoprotein substrates, thus suppressing cell outgrowth. In cancer cells with overexpressed LSD1, the FBXW7 dimerization is blocked by LSD1 binding to FBXW7 in a demethylase-independent manner. FBXW7 self-ubiquitylation will then be triggered, followed by degradation by proteasome as well as lysosome in a p62-dependent pathway. **d** EZH2 is an enzymatic subunit of PRC2, which also includes EED, SUZ12, and RBBP4/7. The SET domain of EZH2 is responsible for the catalyzes the mono-, di-, and trimethylation of H3K27 from the universal methyl donor SAM, after which SAM becomes SAH. EZH2 also has noncanonical functions with its hidden TAD. The EZH2 TAD directly interacts with cMyc and other activators, including p300 and SWI/SNF. GOF gain-of-function, PRC2 polycomb repressive complex 2, SAH S-adenosyl-l-homocysteine, SAM S-adenosyl-l-methionine, TAD transactivation domain

A recent proteogenomic portrait of LSCC suggested that *WHSC1L1* (*NSD3*), but not *FGFR1*, may be the critical driver oncogene within the recurrent focal amplicon (8p11.23).^38^ NSD1, NSD2, NSD3, and ASH1L are four related enzymes in mammals which can synthesize the euchromatin-associated H3K36me2 modification.^286^ NSD3 dimethylates (adds two methyl groups to) the 36th amino acid residue in histone H3 (a lysine residue dubbed H3K36).^287^ This process, in which various chemical groups are covalently added to, or removed from, the DNA bases and the tails of the histones is referred to as epigenetic modifications.^288^ Amplification of *NSD3* and its immediate neighbors (e.g., *FGFR1*), located on the chromosomal region 8p11-12, is one of the frequent molecular alterations in LSCC^289^ and has been implicated in the etiology of LSCC.^290,291^ In contrast to *FGFR1*, gene amplification of *NSD3* correlates strongly with increased mRNA expression.^291^ Accordingly, a recent study has also shown that depletion of NSD3 in the 8p11-12 amplified LSCC cell lines and mouse model significantly attenuated tumor growth.^44^ This study also confirmed the ability of NSD3 to cooperate with SOX2 to transform human tracheobronchial epithelial (AALE) cells which further verified that NSD3 could promote human LSCC tumorgenesis^40^ (Fig. 4a).

Given that currently there is no catalytic inhibitors of NSD3 available in physiological settings, the researchers in that study also found that the four bromodomain inhibitors (BETi) exhibited the highest differential lethality over cells with mutated NSD3.^44^ This clinical actionable vulnerability, accompanied with findings of Li et al.^292^ who used cryo-electron microscopy to solve the structures of normal and oncogenic mutant forms of NSD3 bound to a nucleosome, will certainly provide valuable information for the design and development of drugs for treating LSCC as well as other NSD-driven diseases.

### SETD8

SETD8 (also known as PR-Set7, SET8, and KMT5A) is currently the only known H4K20me1 monomethyltransferase, which is implicated in the regulation of multiple biological activities, including DNA replication, DNA damage repair, cell-cycle progression, and transcription regulation.^293,294^ During mitosis, SETD8 is concentrated in the nucleus during G1 and G2 phases and is degraded through ubiquitination at G1/S transition.^295^ Besides H4K20, SETD8 can also regulate the tumor-suppressor protein p53 and proliferating cell nuclear antigen (PCNA), which are closely related to carcinogenesis.^296–298^ SETD8 is implicated in cancer proliferation, migration, invasiveness, and oncogenesis, associated with a poor outcome.^299,300^

In the study by Lazarus et al.,^45^ *BCL11A*, which encodes a transcriptional regulator, was identified and characterized as a LSCC oncogene. Along with *SOX2*, which was also regarded as an oncogene in LSCC,^40^ this BCL11A-SOX2 transcriptional program provides a potential therapeutic window for LSCC. To disrupt this BCL11A-SOX2 transcriptional program, a Gene Ontology (GO) was performed and the *SETD8* gene is selected, which is regulated by both BCL11A and SOX2.^45^ Knockdown of *SETD* gene could selectively inhibit LSCC tumor growth, but not LUAD cell. Besides, SETD8 inhibition also sensitizes LSCC cell lines to chemotherapy. Collectively, this study highlights the BCL11A-SOX2 transcriptional program as a novel target for LSCC and suggests the monomethyltransferase SETD8 as a potential downstream target^45^ (Fig. 4b).

### LSD1

Lysine-specific demethylase 1 (LSD1, also known as KDM1A, KIAA0601, BHC110, and AOF2) is one of the SOX2-related targets that has been extensively studied. LSD1 is the first identified histone demethylase, which has the dual substrate specificity to catalyze the demethylation of histone 3 lysine 4 (H3K4me1/2) and H3K9me1/2 for transcriptional repression.^301–303^ The expression of LSD1 histone demethylase was reported to be significantly elevated in SOX2-expressing LSCC.^46^ LSCC cell lines with amplified *SOX2* gene are particularly sensitive to LSD1 inactivation, whereas SOX2-negative cells are not. The regulation of *SOX2* gene by LSD1 is directly through the bivalent H3K4 and H3K9 methylations. As a key regulator of *SOX2*, which is a lineage-survival oncogene of LSCC,^40^ LSD1 can serve as a specific and selective target for the treatment of LSCC.

In addition to its demethylase activity, the demethylase-independent activity of LSD1 has also been implicated in carcinogenesis.^47,304,305^ LSD1 can act as a pseudosubstrate of FBXW7. FBXW7 is a typical tumor suppressor that targets many oncoproteins for ubiquitylation and degradation.^261^ FBXW7 dimerization is disrupted by the binding of FBXW7 and LSD1 which promotes FBXW7 self-ubiquitylation and degradation through proteasome and lysosomal pathways, independent of the demethylase activity of LSD1, thus leading to accelerated growth^47^ (Fig. 4c). The discovery of this demethylase-independent activity of LSD1 implicates that the efforts to develop LSD1 inhibitors should be extended to directly target LSD1 rather than just inhibit its demethylase activity, which should harbor broader utility in anticancer therapy.

Currently, many LSD1 inhibitors are tested in phase I/II clinical trials,^306^ although most inhibitors were based on blocking its demethylase activity. However, the ineffectiveness of catalytic inhibition of LSD1 has been noticed in certain cancers.^307,308^ Therefore, targeting LSD1-involved protein interactions with the emerging technologies of PROTACs,^309^ not confined to the inhibition of its demethylate activity, may be a novel anticancer therapy in cancers with LSD1 overexpression like LSCC.

### EZH2

Enhancer of zeste homolog 2 (EZH2) is a histone methyltransferase associated with transcriptional repression.^310^ As an enzymatic subunit of the polycomb repressive complex 2 (PRC2), EZH2 catalyzes the addition of methyl group to histone 3 lysine 27 (H3K27), which serves as an anchor point for the recruitment of additional polycomb group proteins, contributing to formation of a repressive chromatin state.^311,312^ Overexpression of EZH2 is observed in numerous tumor entities^313–318^ and associated with cancer progression and poor outcomes in patients.^319,320^ In LSCC, EZH2 dependency was observed in SOX2 amplified LSCC cell lines.^38^ Accordingly, the inhibition of EZH2 in TNBC could lead to the downregulation of SOX2 expression,^48^ suggesting that the inhibition of EZH2 may be an alternative strategy to depress the expression of SOX2 in LSCC.

EZH2 is usually found in actively proliferating tissues, whereas its homolog EZH1 is present in both dividing and differentiated cells.^321^ Compared to PRC2-EZH2, PRC2-EZH1 demonstrates a lower histone methyltransferase activity, and its knockdown alone does not lead to global reduction of H3K27 methylation.^322^ This suggests that EZH2 plays a predominant role in H3K27 methyltransferase in malignant cells and provides a rationale for the development of EZH2-specific inhibitors. On the other hand, the residual H3K27 after EZH2 inhibition also suggests the rationale for dual EZH1 and EZH2 inhibition. Apart from its catalytic function, EZH2 could also modulate gene expression during carcinogenesis in a PRC2-independent way.^323^ This PRC2-independent functions is associated with other non-PRC2 partners, the interactions with which are often methyltransferase-independent.^324–327^ For example, EZH2 could exert its oncogenic function as a transcriptional coactivator of androgen receptor in cells of castration-resistant prostate cancer.^324^ In acute leukemia, the noncanonical functions of EZH2 were fulfilled by binding cMyc at non-PRC2 targets and using a hidden transactivation domain (TAD) for activator recruitment and gene activation^49^ (Fig. 4d). The discovery of the noncanonical function of EZH2 provides rationale for the development of EZH2 SMIs, without necessarily inhibiting its catalytic function.

Currently, there is only one EZH2 inhibitor tazemetostat approved by FDA for advanced epithelioid sarcoma and follicular lymphoma^328,329^ while other EZH2 inhibitors are still being tested in phase I/II clinical trials. Most EZH2 inhibitors developed are catalytic inhibitors of EZH2 which targets its methyltransferase function. To suppress the multifaceted activities of EZH2, including both the canonical and noncanonical activities, a PROTACs technique-based degrader, MS177, has been shown to be fast-acting and more potent in suppressing tumor growth.^49^ This surely represents a promising therapeutic strategy for the development of EZH2 inhibitors.

## Targeting the immune checkpoint in LSCC

Cancer immunotherapy has emerged as a powerful tool in the armamentarium against cancer, especially for LSCC which is refractory to currently available chemotherapy and targeted therapies. The field of oncology has been revolutionized by the emergence of cancer immunotherapy with significantly prolonged survival of patients in several fatal cancer types. Immunotherapy is increasingly being used as first-line treatment for many cancer indications. The idea of cancer immunotherapy against cancer is to deploy the immune system as a tool to treat neoplastic diseases. The first well-documented attempt to tackle cancer via the immune system dates back to 1890s, when Dr. William Coley, known as the “Father of Cancer Immunotherapy”, injected streptococcal organisms into a patient with inoperable cancer.^330^ We now understand that this effect is achieved by nonspecific immune stimulation, an approach that, while working well, received a lot of criticism at that time. Compared with traditional chemotherapy and targeted therapy, immunotherapy has potential efficacy across the boundaries of histology and driver mutational status and can lead to sustained remissions for those patients who achieve a response with fewer side effects.^24,25,331^ In this part, we will mainly discuss ICB therapies in LSCC. The current application of cancer immunotherapy in LSCC was summarized in Table 1. We will also present new insights into current immunotherapeutic targets as well as new targets for ICB therapies.

**Table 1:** ICB therapies approved by FDA or NMPA for the treatment of LSCC

Drug	Brand name	Developer	Target	Approved treatment options for LSCC	Approval time	Related trial
Nivolumab	Opdivo®	Bristol-Myers Squibb Co.	PD-1	Neoadjuvant treatment with platinum-doublet chemotherapy for adult patients with resectable NSCLC (The first FDA approval of a checkpoint inhibitor for neoadjuvant treatment of lung cancer)	3/4/2022	CHECKMATE-816 (NCT02998528)
				First-line treatment plus ipilimumab and 2 cycles of platinum-doublet chemotherapy for patients with metastatic NSCLC, with no EGFR or ALK genomic tumor aberrations. (FDA)	3/26/2020	CHECKMATE-9LA (NCT03215706)
				First-line treatment plus ipilimumab for patients with metastatic NSCLC whose tumors express PD-L1 (≥1%) with no EGFR or ALK genomic tumor aberrations (The first and currently the only FDA approval of a checkpoint inhibitor combination for the treatment of NSCLC)	3/15/2020	CHECKMATE-227 (NCT02477826)
				Second-line treatment for patients with metastatic squamous NSCLC whose disease progressed during or following platinum-containing chemotherapy (FDA)	3/4/2015	CHECKMATE-017 (NCT01642004)
Pembrolizumab	Keytruda®	Merck & Co. Inc.	PD-1	First-line treatment for patients with stage III NSCLC who are not candidates for surgical resection or definitive chemoradiation or metastatic NSCLC. Patients’ tumors must have EGFR or ALK genomic aberrations and express PD-L1 (TPS ≥ 1%) (FDA)	4/11/2019	KEYNOTE-042 (NCT02220894)
				First-line treatment with carboplatin and either paclitaxel or nab-paclitaxel for metastatic squamous NSCLC (FDA)	10/30/2018	KEYNOTE-407 (NCT02775435)
				First-line treatment for patients with metastatic NSCLC whose tumors have high PD-L1 expression (TPS ≥ 50%), with no EGFR or ALK genomic tumor aberrations, and no prior systemic chemotherapy treatment for metastatic NSCLC (The first FDA approval of a checkpoint inhibitor for first-line treatment of lung cancer)	10/24/2016	KEYNOTE-024 (NCT02142738)
				Second-line treatment for patients with metastatic NSCLC whose tumors express PD-L1 (TPS ≥ 1%), with disease progression on or after platinum-containing chemotherapy. (FDA)	10/24/2016	KEYNOTE-024 (NCT02142738)
Cemiplimab	Libtayo®	Regeneron Pharmaceuticals, Inc.	PD-1	First-line treatment for patients with advanced NSCLC (locally advanced who are not candidates for surgical resection or definitive chemoradiation or metastatic) whose tumors have high PD-L1 expression (TPS ≥ 50%), with no EGFR, ALK or ROS1 genomic tumor aberrations (FDA)	2/22/2021	EMPOWER-Lung 1 (NCT03088540)
Atezolizumab	Tecentriq®	Genentech, Inc.	PD-L1	Adjuvant treatment following resection and platinum-based chemotherapy in patients with stage II to IIIA NSCLC whose tumors have PD-L1 expression on ≥1% of tumor cells (The first FDA approval of a checkpoint inhibitor for adjuvant treatment of lung cancer)	10/15/2021	IMpower010 (NCT02486718)
				First-line treatment for adult patients with metastatic NSCLC whose tumors have high PD-L1 expression (TC ≥ 50% or IC ≥ 10%), with no EGFR or ALK genomic tumor aberrations (FDA)	5/18/2020	IMpower110 (NCT02409342)
				Second-line treatment for patients with metastatic NSCLC whose disease progressed during or following platinum-containing chemotherapy (FDA)	10/18/2016	OAK (NCT02008227) POPLAR (NCT01903993)
Durvalumab	Imfinzi®	AstraZeneca Inc.	PD-L1	Treatment for patients with unresectable stage III NSCLC whose disease has not progressed following concurrent platinum-based chemotherapy and radiation therapy (FDA)	2/16/2018	PACIFIC (NCT02125461)
Ipilimumab	Yervoy®	Bristol-Myers Squibb Co	CTLA-4	First-line treatment plus nivolumab and 2 cycles of platinum-doublet chemotherapy for patients with metastatic NSCLC, with no EGFR or ALK genomic tumor aberrations (FDA)	3/26/2020	CHECKMATE-9LA (NCT03215706)
				First-line treatment plus nivolumab for patients with metastatic NSCLC whose tumors express PD-L1 (≥1%) with no EGFR or ALK genomic tumor aberrations (The first and currently the only FDA approval of a checkpoint inhibitor combination for the treatment of NSCLC)	3/15/2020	CHECKMATE-227 (NCT02477826)
Sintilimab	Tyvyt®	Innovent Biologics (Suzhou) Co. Ltd.	PD-1	First-line treatment with gemcitabine and platinum for patients with locally advanced or metastatic squamous NSCLC (NMPA)	6/1/2021	ORIENT-12 (NCT03629925)
Camrelizumab	AiRuiKa®	Jiangsu Hengrui Pharmaceuticals	PD-1	First-line treatment with carboplatin and paclitaxel for patients with locally advanced or metastatic squamous NSCLC (NMPA)	12/10/2021	CameL-sq (NCT03668496)
Tislelizumab	BaiZeAn®	BeiGene	PD-1	Second-line or third-line treatment for patients with locally advanced or metastatic NSCLC with disease progression during or following treatment with at least one platinum-containing regimen	1/5/2022	RATIONALE 303 (NCT03358875)
				First-line treatment with carboplatin and either paclitaxel or nab-paclitaxel for patients with locally advanced or metastatic squamous NSCLC (NMPA)	1/14/2021	RATIONALE 307 (NCT03594747)
Sugemalimab	Cejemly®	CStone Pharmaceuticals	PD-L1	First-line treatment with carboplatin and paclitaxel for patients with metastatic squamous NSCLC (NMPA)	12/21/2021	GEMSTONE-302 (NCT03789604)

*ALK* anaplastic lymphoma kinase, *CTLA-4* cytotoxic T-lymphocyte antigen 4, *EGFR* epidermal growth factor receptor, *FDA* Food and Drug Administration, *ICB* immune-checkpoint blockade, *IC* *≥* *10%* PD-L1 stained tumor-infiltrating immune cells covering ≥10% of the tumor area, *LSCC* lung squamous cell carcinoma, *NMPA*, National Medical Products Administration, *NSCLC* non-small-cell lung cancer, *PD-1* programmed cell death 1, *PD-L1* programmed cell death ligand 1, *TC* *≥* *50%* PD-L1 stained ≥50% of tumor cells, *TPS* tumor proportion score.

® Registered sign.

### ICB therapy

Immune-checkpoint blockade is one of the most promising approaches to activating antitumor immunity. The immune-checkpoint pathways are involved in the major mechanisms underlying tumor immune evasion. Physiologically, these immunosuppressive signaling pathways play important roles in maintaining self-tolerance to prevent autoimmunity, limit immune-mediated tissue damage, and control the resolution of inflammation.^332,333^ Cancer cells may take advantage of these immune checkpoints to disguise themselves from body immune system.^334,335^ Among these immune checkpoints, cytotoxic T-lymphocyte antigen 4 (CTLA-4) and PD-1/PD-L1 axis are the most potent examples of T-cell immune-checkpoint molecules. The ICB therapies which were approved by FDA or National Medical Products Administration (NMPA) for LSCC are summarized in Table 1.

### CTLA-4: the first clinically targeted immune-checkpoint receptor

CTLA-4 is a homolog of CD28 and binds both B7-1 (also known as CD80) and B7-2 (also known as CD86) with much higher affinity than CD28.^336–339^ The CTLA-4 and CD28 genes are located in the same region of chromosome 2 (2q33.2) and are expressed by both CD4^+^ and CD8^+^ T cells with opposing functions in T-cell activation.^337,339^ Through interacting with a pair of ligands (B7-1 and B7-2) expressed on antigen-presenting cells (APCs), including macrophages, dendritic cells (DCs) and B cells, CD28 mediates T-cell activation by co-stimulating T-cell receptor (TCR) signaling while the interaction of the ligands with CTLA-4 serves to inhibit T-cell response.^340^ These regulatory effects of CTLA-4 mainly restrict the expansion of CD4^+^ helper T cells while boosting regulatory T cells (Tregs),^334,341^ thus leading to a pro-tumor immunosuppressive phenotype.^342^

The recognition of CTLA-4 as a negative regulator of T-cell activation makes antagonizing CTLA-4 a reasonable method to enhance the antitumor immunity of T cells.^343^ Initial preclinical studies found that CTLA-4 blockade enhanced antitumor immunity and caused regression of immunogenic tumors without inducing substantial autoimmunity.^344,345^ Based on these preclinical findings, several clinical trials have been conducted to evaluate the therapeutic efficacy of CTLA-4 antibodies in tumors,^346–349^ which finally led to the FDA approval of ipilimumab by FDA for the treatment of advanced melanoma. However, the impressive effects of ipilimumab in melanoma patients did not proceed in renal cell carcinoma,^350^ NSCLC,^351^ small-cell lung cancer^352^ and prostate cancer.^353^ Another CTLA-4-blocking antibody, tremelimumab, has not received FDA approval since it did not improve survival compared to chemotherapy in metastatic melanoma.^354^ As the first immune-checkpoint inhibitor, ipilimumab is also currently the only CTLA-4-blocking antibody that has gained approval for anticancer treatment. No CTLA-4 inhibitors have been approved as monotherapy or in combination therapy with chemotherapy for the treatment of NSCLC (Table 1).

**Fig. 5:**
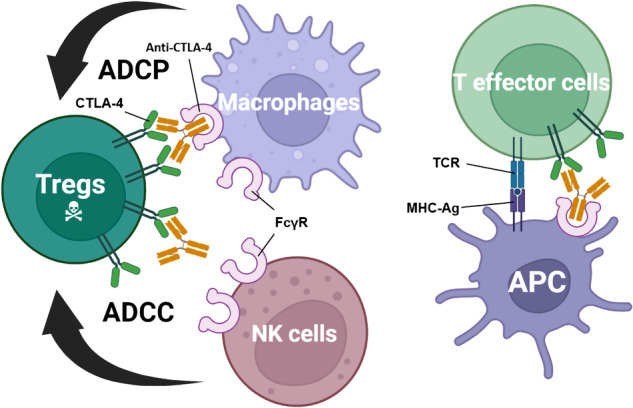
Roles of Fcγ receptors in anti-CTLA-4 function. Selective deletion of Tregs in the tumor microenvironment results in tumor immunity (left). Expressing higher levels of CTLA-4 than effector T cells, intratumoral Tregs are selectively depleted through ADCP by macrophages and/or ADCC by NK cells. In T-effector cells, T-cell activity is enhanced by the recognition of MHC-Ag by the TCR in the presence of an anti-CTLA-4 antibody that had co-engaged with FcγR on APCs (right). ADCC antibody-dependent cellular cytotoxicity, ADCP antibody-dependent cellular phagocytosis, APC antigen-presenting cells, MHC-Ag major histocompatibility complex-antigen peptide complexes major histocompatibility complex-antigen peptide complexes, NK cells natural killer cells, TCR T-cell receptor

The fact that anti-CTLA-4 antibodies are capable to induce long-term immunity in cancer patients demonstrates that CTLA-4 remains an important immunotherapy target.^355,356^ Nevertheless, CTLA-4-targeting inhibitors have not reached its full potential, as evidenced by high rates of immunotherapy-related adverse effects (irAEs) and relatively low response rates. The strong irAEs of ipilimumab limit the doses tolerated by cancer patients. Both anti-PD-1/PD-L1 antibodies and anti-CTLA-4 antibodies have irAEs, while the effects of anti-CTLA-4 therapy are generally more severe.^357–360^ The dose-limiting toxicity of ipilimumab presented an opportunity of developing the next-generation molecules with wider therapeutic window.^361–363^ Recently, additional mechanisms were raised to explain the immunotherapeutic effects of anti-CTLA-4 mAbs, including depletion of regulatory T cells (Tregs) in TME.^341,364–367^ According to Du et al.,^368^ ipilimumab remains full activity without blocking B7-CTLA-4 interaction. In their studies, the humanized antibodies they developed without blockade of the B7-CTLA-4 interaction were as effective as ipilimumab at causing rejection of cancer. To further confirm that this tumor rejection was induced by Tregs depletion through antibody-dependent cellular cytotoxicity (ADCC), concurrent administration of anti-FcR antibodies treatment completely abolished the anticancer effect of ipilimumab. Collectively, these findings suggest that the selective Treg depletion in the tumors may be the primary mechanism of antitumor effect of anti-CTLA-4 antibody rather than the blockade of B7-CTLA-4 interactions^369^ (Fig. 5).

Many new types of anti-CTLA-4 antibodies have been developed to increase antitumor effect, reduce side effects, or both. Increasing the ability of Fc to bind to FcR is one of the strategies to enhance the antitumor effect which can be achieved through a non-fucosylated derivative of ipilimumab (BMS986218) or an engineered Fc variant of an anti-CTLA-4 antibody (AGEN-1181or its mouse surrogate).^370^ The next-gen anti-CTLA-4 mAb, ONC-392, which effectively and selectively eliminates Tregs, has been granted Fast Track designation granted by FDA for monotherapy in PD-(L)1-resistant NSCLC.^371^ Different from other anti-CTLA-4 mAbs being tested, the pH-sensitivity nature of ONC-392 avoids antibody-triggered lysosomal degradation of CTLA-4, thereby reducing toxicity and exerting its anticancer potential.^363^ ONC-392 is currently being evaluated in Phase I clinical trial (PRESERVE-001; NCT04140526) for advanced solid tumors and NSCLC. An additional approach to moderate the adverse event profile of anti-CTLA-4 is to limit the CTLA-4 blockade within the tumor. For example, a “proform” of ipilimumab (BMS-986249) was synthesized, which was designed to remain inert in the periphery, but have activity restored when unmasked by tumor-associated proteases.^370^ Another approach is to generate a pH-selective form of ipilimumab, which could preferentially and reversibly target the acidic TME over the neutral periphery.^362^

### PD-1 axis

PD-1 axis was the second immune-checkpoint pathway targeted for ICB therapy. In 2014, fully humanized anti-PD-1 mAbs pembrolizumab and nivolumab became the first PD-1 targeted therapeutics approved by FDA for refractory and advanced melanoma.^357,372–374^ Although anti-PD-(L)1 therapy entered the market later than anti-CTLA-4 therapy, PD (L)-1 blockade have shown broader clinical utility than anti-CTLA-4 treatment. For LSCC, a number of anti-PD-(L)1 therapeutics have been approved by FDA and NMPA as monotherapy or in combination therapy with chemotherapy (Table 1).

PD-1 was first identified as a putative mediator of apoptosis in 1992,^375^ and its role in maintaining peripheral tolerance by serving as a negative regulator of immune responses was elucidated in 1999 when Nishimura et al.^376^ found that PD-1-deficient mice developed a late onset of lupus-like autoimmune disease. Nearly at the same time, Dong et al.^377^ revealed a new member of the B7 family which might be involved in the negative regulation of cell-mediated immune responses. In the next year, this new member of the B7 family was confirmed to be the ligand of PD-1 (PD-L1) and an inhibitor of T-cell activation.^335^ PD-L2, a second ligand with higher affinity for PD-1, was also identified.^378,379^ Subsequent work found out that PD-L2 could have both co-stimulatory and co-inhibitory functions depending on the receptor and context.^380^ After being implicated in the negative regulation of T cells, the PD-1 axis was regarded as an active target of developing anticancer therapies. Multiple preclinical studies have showed that the PD-1 axis in the tumor causes the resistance to immune-mediated cytolysis, while blocking PD-L1 or PD-1 with specific mAbs in tumors could reverse tumors’ inherent resistance to cytotoxicity by T cells.^381–384^ However, solely blocking PD-L2 did not demonstrate any antitumor effect.^385^ Following the success of preclinical studies, mAbs targeting the PD-1 axis were designed and showed remarkable efficacy in clinical trials. In a head-to-head comparison for PD-L1 expressing advanced NSCLC, monotherapy with the PD-1 inhibitor pembrolizumab showed significantly better OS and lower incidence of adverse events than chemotherapy.^386,387^ The PD-L1 inhibitor atezolizumab also resulted in significantly longer OS than platinum-based chemotherapy in NSCLC patients with high PD-L1 expression.^27,388^

As studies in immunotherapy increase, difference between the clinical effect of anti-PD-1 and anti-PD-L1 has been reported. Such disparities have drawn the attention of clinicians and a better understanding of this discrepancy may guide us for a better administration of these drugs. Currently there are no head-to-head comparisons of anti-PD-1 mAbs and anti-PD-L1 mAbs in clinical trials. In a systematic review and meta-analysis, Duan et al.^389^ adjusted indirect comparisons based on a well-designed mirror principle to minimize the potential bias and found out that anti-PD-1 mAbs appeared to exhibit significantly greater OS compared with anti-PD-L1 with a comparable safety profile in patients with solid tumors. The possible reason for the improved efficacy of anti-PD-1 mAbs compared with anti-PD-L1 mAbs may come from the mechanisms of PD-1 and PD-L1 blockade in anticancer therapy. Anti-PD-1 mAbs can bind to PD-1 and further block the interaction between PD-1 and its ligands (PD-L1 and PD-L2), while the PD-1/PD-L2 axis remains intact and exerts its immune suppressive functions when PD-L1 is blocked by anti-PD-L1 mAbs. Nevertheless, the blockade of PD-1 may shift the balance of the binding of PD-L2 with its other partner, repulsive guidance molecule b (RGMb), which can lead to pneumonitis.^380^ This is also confirmed by the fact that patients treated with PD-1 inhibitors have a higher incidence of pneumonitis than patients who received PD-L1 inhibitors.^390,391^

Although great success has been achieved in the treatment of LSCC with the advent of PD-1 axis inhibitors, the ORR of PD-1 axis inhibitor in the treatment of advanced NSCLC is ~30%.^27,387^ Therefore, it is of utmost importance for the establishment of effective biomarkers for predicting the efficacy of anti-PD-1 axis agents. The assessment of PD-L1 expression on tumor cells is a logical biomarker for the prediction of treatment response to anti-PD-1 or anti-PD-L1 therapies. A real-world study in China has found out that LSCC patients were associated with higher incidence rate of positive PD-L1 expression, suggesting a benefit of using ICIs in LSCC patients.^392^ Although PD-L1 immunohistochemistry (IHC) plays an important role in patient stratification in clinical trials of anti-PD-1 or anti-PD-L1 therapies, it has poor reliability as a biomarker for anti-PD-1 or anti-PD-L1 therapies, as patients with negative PD-L1 expression can still benefit from anti-PD-1 or anti-PD-L1 therapies.^393–395^ Beyond PD-L1 expression, several other biomarkers have also successively predicted the efficacy of ICB therapy to certain extent. Among them, tumor mutational burden (TMB), gene expression profiling (GEP), and multiplex immunohistochemistry/immunofluorescence (mIHC/IF) are mostly used.^396^ Due to the lack of accurate assessment of response, future improvements in diagnostic accuracy may be achieved through a multiple incorporation of existing markers and newly discovered markers.^396–400^

### LAG3

Lymphocyte activating gene 3 (LAG3, also known as CD223), first discovered in 1990,^401^ is a transmembrane molecule that is expressed on CD4^+^ and CD8^+^ T cells, natural killer T (NKT) cells, natural killer (NK) cells, plasmacytoid dendritic cells (pDCs) and Tregs.^402,403^ The LAG3 gene is located on human chromosome 12 (12p13.31), adjacent to the coding region of CD4.^404^ The LAG3 protein and CD4 protein share approximately 20% similarities in their amino acid sequences, which is mostly pronounced on their extracellular regions.^404,405^ Due to this similarity in extracellular structures, like CD4, LAG3 can also bind to major histocompatibility complex class II (MHC-II) proteins, but with higher affinity, which is also the canonical ligand for LAG3.^406^ Once LAG3 binds to MHC-II proteins, the inhibitory signals are transmitted through its cytoplasmic domain, thereby downregulating T-cell function.^407^ Several other ligands were also found to interact with LAG3, including Galectin-3^408^ (Gal-3), liver sinusoidal endothelial cell lectin^409,410^ (LSECtin), and fibrinogen-like protein 1^411^ (FGL1).

The fact that LAG3-deficient T cells show enhanced homeostatic expansion suggests the inhibitory role of LAG3 in immune responses.^412,413^ LAG3 is co-expressed with other inhibitory receptors, such as PD-1, on CD8^+^ tumor antigen-specific T cells under chronic tumor antigen stimulation, which leads to T-cell exhaustion.^403,414^ LAG3 expression was also confirmed to play an important role in supporting Tregs activity.^402^ In intratumoral Tregs, LAG3 is expressed at a higher level than in Tregs found in peripheral or normal tissue.^415,416^ Multiple LAG3-modulating candidates have been developed, including LAG3-inhibiting antibody and LAG3 fusion protein.^417^ However, LAG3 monotherapy in several mouse models has shown limited antitumor effect with slightly reduced tumor growth, whereas LAG3/PD-1 co-blockade has shown much stronger synergistic antitumor effects.^418–422^ Several anti-LAG3 antibodies are currently being evaluated in clinical trials, in which relatlimab is the furthest along in clinical development among all the anti-LAG3 mAb. On March 18, 2022, the combination therapy of relatlimab and nivolumab was approved by FDA for the treatment of unresectable or metastatic melanoma, making LAG3 the third FDA-approved immune checkpoint that was approved by FDA after CTLA-4 and PD-1 axis.^423,424^ This approval of LAG3 mAb marks an exciting beginning for this inhibitory receptor but many aspects of its biological functions still remain enigmatic. New ligands of LAG3 are still emerging and the effects of LAG3 on immune cells remain to be fully characterized.^417^ For LSCC, several anti-LAG3 antibodies and bispecific antibodies are being evaluated in phase I and phase II clinical trials (Table 2).

**Table 2:** Ongoing ICB therapy-based mono- or combination clinical trials for medications that have not be approved in treating LSCC

Target	Agents	Combinations	Registration number	Trial name	Phase	Enrollment	Target patients	Status
PD-1	Toripalimab	Toripalimab with chemo	NCT04158440	/	III	406	Resectable stage II–III NSCLC	Recruiting
		Adjuvant toripalimab and chemo	NCT04772287	LungMate-008	III	341	Stage II-IIIB(N2) NSCLC without EGFR/ALK Mutation	Not yet recruiting
	Zimberelimab	Zimberelimab monotherapy or plus AB154	NCT04736173	/	III	625	Locally advanced or metastatic PD-L1-selected NSCLC	Recruiting
	Penpulimab	Neoadjuvant penpulimab plus chemotherapy followed by adjuvant penpulimab	NCT04846634	ALTER-L043	II	90	Resectable IIB-IIIB (N2) NSCLC	Not yet recruiting
	Envafolimab	Envafolimab plus chemotherapy and recombinant human endostatin	NCT05243355	/	II	46	Advanced (Stage IIIB-IV) LSCC	Recruiting
PD-L1	Avelumab	Avelumab monotherapy	NCT02576574	JAVELIN Lung 100	III	1224	Recurrent or metastatic PD-L1-selected NSCLC	Active, not recruiting
	SHR1701	SHR1701 with or without chemo	NCT04580498	/	II	122	Unresectable Stage III NSCLC	Not yet recruiting
	SHR1316	Neoadjuvant SHR1316 and chemo followed by adjuvant SHR1316	NCT04316364	/	Ib/III	537	Resectable stages II, IIIA, or selected IIIB NSCLC	Recruiting
	TQB2450	TQB2450 with or without anlotinib	NCT04325763	/	III	315	Locally advanced or unresectable, stage III NSCLC without progression after prior concurrent/sequential chemoradiotherapy	Recruiting
CTLA-4	Tremelimumab	Tremelimumab plus durvalumab and chemo	NCT03164616	POSEIDON	III	1193	Metastatic NSCLC	Recruiting
	BMS986218	BMS986218 monotherapy or with ipilimumab	NCT03110107	/	I/IIa	390	Advanced solid tumors	Recruiting
	Quavonlimab	Quavonlimab with pembrolizumab	NCT03516981	KEYNOTE-495	II	318	Advanced NSCLC	Active, not recruiting
	ONC-392	ONC-392 monotherapy or with pembrolizumab	NCT04140526	PRESERVE-001	Ia/Ib	413	Advanced solid tumors and NSCLC	Recruiting
LAG3	Relatlimab	Neoadjuvant nivolumab with or without relatlimab	NCT04205552	NEOpredict	II	60	Clinical stages IB, II and selected stage IIIA NSCLC	Recruiting
		Relatlimab plus nivolumab and chemo	NCT04623775	/	II	520	Metastatic or recurrent NSCLC	Recruiting
	IBI110	IBI110 with or without sintilimab	NCT04085185	/	I	268	Advanced malignant tumors	Recruiting
TIGIT	Tiragolumab	Tiragolumab and atezolizumab	NCT04294810	SKYSCRAPER-01	III	635	Locally advanced unresectable or metastatic PD-L1-selected NSCLC	Recruiting
		Tiragolumab and atezolizumab	NCT04513925	SKYSCRAPER-03	III	800	Locally advanced, unresectable stage III NSCLC after at least two cycles of platinum-based cCRT without radiographic disease progression.	Recruiting
	Ociperlimab	Ociperlimab and tislelizumab	NCT04746924	/	IIIIII	605	Locally advanced unresectable or metastatic PD-L1-selected NSCLC	Recruiting
	Domvanalimab	Domvanalimab and durvalumab	NCT05211895	PACIFIC-8	III	860	locally advanced, unresectable Stage III NSCLC without progression following definitive platinum-based cCRT.	Recruiting
	Vibostolimab	Vibostolimab and pembrolizumab	NCT04738487	KEYVIBE-003	III	1246	Metastatic PD-L1-selected NSCLC	Recruiting
TIM3	BGB-A425	BGB-A425 and tislelizumab	NCT03744468	/	I–II	162	Advanced solid tumors	Recruiting
	MBG453	MBG453 monotherapy or with PDR001	NCT02608268	/	I-Ib/II	252	Advanced solid tumors	Active, not recruiting
4-1BB	PF-05082566	PF-05082566 and avelumab	NCT02554812	JAVELIN Medley	II	398	Advanced solid tumors	Active, not recruiting
OX40	BMS-986178	BMS-986178 monotherapy or with nivolumab or ipilimumab	NCT02737475	/	I/IIa	166	Advanced solid tumors	Completed
CTLA-4 & PD-L1 (Bispecific antibody)	KN046	KN046 and chemo	NCT04474119	ENREACH-L-01	III	482	Advanced LSCC	Active, not recruiting
		KN046 and Lenvatinib	NCT05001724	/	II/III	522	Advanced NSCLC after failure of prior anti-PD-(L)1 agent	Recruiting
CTLA-4 & PD-1 (Bispecific antibody)	AK104	AK104 and docetaxel	NCT05215067	/	II	40	Advanced NSCLC after the failure of prior platinum doublet chemotherapy and anti-PD-1/PD-L1 agent	Recruiting
		Neoadjuvant AK104 and adjuvant AK104 plus chemotherapy with or without radiotherapy	NCT05377658	/	II	32	Resectable stage II-IIIA NSCLC	Not yet recruiting
		AK104 and anlotinib	NCT04544644	/	II	30	Advanced NSCLC	Not yet recruiting
PD-L1 & LAG3 (Bispecific antibody)	IBI323	IBI323 with or without chemo	NCT04916119	/	I	322	Advanced solid tumors	Recruiting
PD-1 & TIM3 (Bispecific antibody)	AZD7789	AZD7789 monotherapy	NCT04931654	/	I/IIa	81	Stage IIIB to IV NSCLC	Recruiting
PD-1 & VEGF (Bispecific antibody)	AK112	AK112 with chemo	NCT04736823	/	II	206	Stage IIIB/C or IV NSCLC	Recruiting
LSD1	CC-90011	CC-90011 with nivolumab	NCT04350463	/	II	92	ES SCLC and advanced LSCC	Active, not recruiting
EZH2	CPI-1205	CPI-1205 with ipilimumab	NCT03525795	ORIOn-E	I/II	24	Advanced solid tumors	Completed
DNMT	Guadecitabine	Guadecitabine plus pembrolizumab and mocetinostat	NCT03220477	/	I	28	Advanced NSCLC after failure of prior anti-PD-(L)1 agent	Active, not recruiting
	Decitabine	Decitabine plus pembrolizumab and tetrahydrouridine	NCT03233724	/	I/II	85	Locally advanced or metastatic NSCLC, esophageal carcinomas, or pleural mesotheliomas	Recruiting
	Azacitidine	Azacitidine plus pembrolizumab	NCT02546986	/	II	100	Advanced NSCLC	Active, not recruiting

*4-1BB* tumor necrosis factor receptor superfamily member 9, *ALK* anaplastic lymphoma kinase, *cCRT* concurrent chemoradiation therapy, *CTLA-4* cytotoxic T-lymphocyte antigen 4, *EGFR* epidermal growth factor receptor, *LAG3* Lymphocyte activating gene 3, *LSCC* lung squamous cell carcinoma, *NMPA* National Medical Products Administration, *NSCLC* non-small-cell lung cancer, *OX40* tumor necrosis factor receptor superfamily member 4, *PD-1* programmed cell death 1, *PD-L1* programmed cell death ligand 1, *SCLC* small-cell lung cancer, *TIGIT* T-cell immunoreceptor with immunoglobulin and immunoreceptor tyrosine-based inhibitory motif domain, *TIM3* T-cell immunoglobulin 3, *TPS* tumor proportion score, / not found

### Other targets for ICB

More negative regulators of T-cell activation have been discovered which are potential targets for ICB, including T-cell immunoreceptor with immunoglobulin and immunoreceptor tyrosine-based inhibitory motif domain (TIGIT), T-cell immunoglobulin 3 (TIM3), V-domain immunoglobulin suppressor of T-cell activation (VISTA).^370^

TIGIT is a member of the immunoglobulin superfamily and was first identified in 2009.^425^ Highly expressed on human and murine tumor-infiltrating T cells,^426^ dual PD-1/PD-L1 and TIGIT blockade is a promising combination immunotherapy for cancer. Co-targeting of TIGIT with PD-1 axis is supported by preclinical studies, which demonstrated a synergistic effect in augmenting proliferation and function of antitumor CD8^+^ T cells than that shown in each single blockade.^426–428^ Tiragolumab is the first anti-TIGIT mAb tested in a phase II study. In the phase II CITYSCAPE study,^429^ tiragolumab plus atezolizumab as a first-line treatment for PD-L1-positive NSCLC have shown significantly improved efficacy compared with atezolizumab alone. Despite the success in this phase II study, the phase III SKYSCRAPER-01 study, which evaluated tiragolumab plus atezolizumab for PD-L1-high metastatic NSCLC, did not meet its co-primary endpoint of PFS while the other co-primary endpoint of OS was immature.^430^ Despite this discouraging news, it is also possible that this combination of immunotherapy may have benefits in long-term efficacy indicators like OS, which has been confirmed in previous immunotherapy clinical trials.^431^ Before the results of OS came out, it might be too early to judge this combination therapy. Currently, three other combination therapies of anti-TIGIT agents and anti-PD-1 axis agents are being evaluated in phase III clinical trials (Table 2).

TIM3 was originally found to be expressed on differentiated Th1 cells, which has also been defined as a marker for terminally differentiated effector Th1 cells.^432,433^ There is a firm connection between elevated TIM3 expression and exhausted CD8^+^ T cell.^434–436^ This elevated expression level of TIM3 in exhausted T cells is also associated with PD-1 expression, suggesting a correlation between TIM3 and PD-1 in T-cells exhaustion.^437–439^ Most clinical trials of TIM3 inhibitors are assessing the efficacy of the combination of TIM3 inhibitors and anti-PD-1 axis mAbs (e.g., NCT03680508, NCT03099109).

Like TIM3, VISTA (also known as PD-1H, B7-H5) is also a promising target for combination immunotherapy.^440^ VISTA shares significant sequence homology with the B7 family ligands PD-L1 and PD-L2 and imposes quiescence on mammalian myeloid and naïve T cells.^441,442^ The interaction of VISTA and its ligand P-selectin glycoprotein ligand 1 (PSGL-1) is governed by pH, selectively at acidic pH such as that found in TME.^443^ Most antibodies that target VISTA are being evaluated in preclinical studies. Only a few anti-VISTA drugs are currently being assessed in phase I studies (e.g., NCT05082610, NCT04564417).

Activating receptors on T cells have also been extensively studied as targets for immunotherapy, including inducible co-stimulator (ICOS, also known as CD278), tumor necrosis factor receptor superfamily member 4 (TNFRSF4, also known as CD134, OX40), tumor necrosis factor receptor superfamily member 9 (TNFRSF9, also known as 4-1BB).^444^ However, the distinct nature of agonist antibodies targeting immune co-stimulatory receptors rendered them unique among other antibody therapies in cancer.^445^ Some next-generation approaches, such as recombinant ligands and bispecific antibodies, may help unlock the full therapeutic potential of such targets.

## Multi-target combination therapeutic strategies

### CTLA-4 and PD-1 axis

The distinct functions of CTLA-4 and PD-1 axis are reflected in the different toxicity seen in their respective knockout mouse models. Mice lacking the CTLA-4 gene developed lymphoproliferative diseases and died by 3–4 weeks of age,^446,447^ whereas mice lacking PD-1 had more limited and variable, model-dependent autoimmunity, including glomerulonephritis, arthritis and cardiomyopathy.^376,448–450^ Spatially, CTLA-4 regulation occurs primarily within lymphoid organs, whereas PD-1 limits T-cell activation locally within peripheral tissues.^451,452^ Temporally, PD-1 acts later during T-cell activation for long-term tolerance. The distinct functions of CTLA-4 and PD-1 axis provide a rationale for the combination therapy of CTLA-4 and PD-1 axis blockade. Combinations of anti-CTLA-4 and anti-PD-1, or anti-CTLA-4 and anti-PD-L1, have shown improved efficacy than either agent alone in clinical trials or preclinical models.^358,453–455^ In NSCLC, based on the results of checkmate 227 clinical trial,^30^ nivolumab plus ipilimumab has been approved by FDA for the first-line treatment of patients with tumors expressing PD-L1(≥1%), which was also the first chemotherapy-free regimen for NSCLC. Besides, nivolumab plus ipilimumab and two cycles of platinum-doublet chemotherapy is also FDA-approved for the first-line treatment of advanced NSCLC, regardless of tumor PD-L1 expression.^31^

Despite the success of nivolumab plus ipilimumab in the treatment of NSCLC, there were also negative results from clinical trials evaluating the combination ICB therapy. In the phase III MYSTIC study, durvalumab plus tremelimumab did not significantly improve OS or PFS compared with chemotherapy in metastatic NSCLC.^456^ For advanced, pretreated, immune-checkpoint inhibitor-naive LSCC, the addition of ipilimumab to nivolumab did not improve outcomes.^457^ These results demonstrate the need for a better mechanistic understanding of the crosstalk among anti-PD-1, anti-PD-L1, and anti-CTLA-4. The cis-PD-L1/CD80 interactions were found to have implications in the synergy of anti-PD-L1 and anti-CTLA-4 combination therapy.^458,459^ Recognized as the ligands of PD-1 and CD28/CTLA-4 respectively, PD-L1 and CD80 were also found to interact with each other.^460,461^ Recent studies reported that PD-L1 and CD80 could heterodimerize in cis when these molecules are overexpressed on the same cell.^458,462^ This PD-L1:CD80 cis-heterodimerization could inhibit both PD-L1:PD-1 and CD80:CTLA-4 interactions through distinct mechanisms while preserving the ability of CD80 to activate the T-cell co-stimulatory receptor CD28. Therefore, by disrupting PD-L1:CD80 heterodimers, anti-PD-L1 mAbs licenses high-avidity CD80:CTLA-4 interactions which triggers Treg-mediated depletion of CD80 from APCs and inhibits CD28 co-stimulation.^458^ Since this CD80 depletion by anti-PD-L1 is CTLA-4 dependent and can be reversed by CTLA-4 blockade,^341,463^ it provides a rationale for co-blocking PD-L1 and CTLA-4 in cancer immunotherapy. In another study, Tekguc et al.^464^ also found that the Treg-mediated depletion of CD80 from APCs via CTLA-4-dependent trogocytosis can also increase free PD-L1 available for the inhibition of PD-1 expressing effector T cells. Therefore, the combination of blocking CTLA-4 and PD-1 axis may synergistically hinder this Treg-mediated immunosuppression and enhance antitumor efficacy.

Some bispecific antibodies, capable of simultaneously binding PD-1/PD-L1 and CTLA-4 with high affinity, are also being evaluated in ongoing clinical trials (Table 2).^465,466^ KN046, a novel bispecific antibody that blocks PD-L1 interaction with PD-1 and CTLA-4 interaction with CD80/CD86, was well tolerated and effective in treating advanced NSCLC, with promising PFS and OS benefits in LSCC.^465^ Pivotal Phase III clinical trials in advanced unresectable or metastatic LSCC is currently ongoing for this bispecific antibody (NCT04474119).

### ICB and PI3K pathway inhibition

**Fig. 6:**
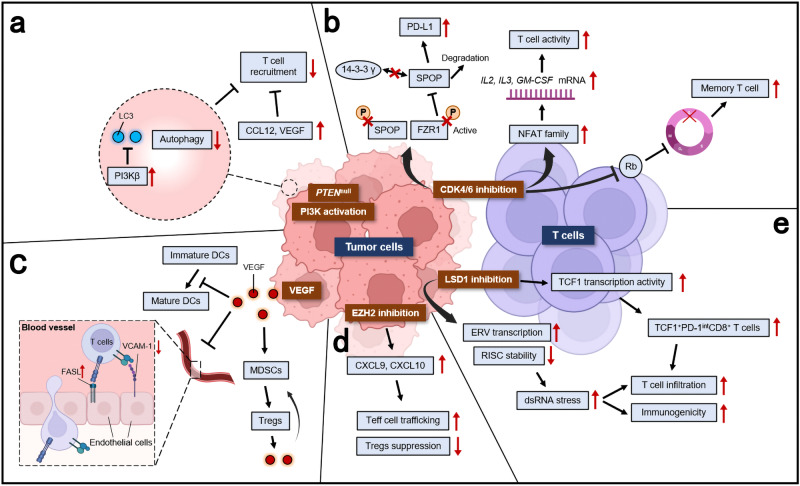
Impact of oncogenic signaling on tumor immune response. **a** Loss of PTEN protein function and improper PI3K activation inhibit efficient LC3 lipidation, which further promote resistance to T-cell-mediated killing by inhibiting autophagy. PTEN loss could also induce expression of immunosuppressive cytokines, including CCL12 and VEGF. **b** CDK4/6 inhibition enhances T-cell activation through the derepression of NFAT family proteins and their target genes, which encodes critical regulators of T-cell function. CDK4/6 inhibition could also induce Rb-mediated G1-arrest and promote the phenotypic and functional acquisition of immunologic T-cell memory. Besides, the PD-L1 protein stability is regulated by the CDK4-SPOP-FZR1 signaling pathway. Physiologically, PD-L1 protein stability is negatively regulated through phosphorylating its upstream physiological E3 ligase SPOP. This phosphorylation promotes SPOP binding to 14-3-3γ, which subsequently disrupts FZR1-mediated destruction of SPOP. The inhibition of CDK4/6 inhibits the phosphorylation of SPOP, thus promoting its degradation by FZR1, thus increasing PD-L1 protein levels. **c** Tumor-derived VEGF limits NF-κB activation in immature DCs, which in turn leads to defective functional maturation of DCs and insufficient induction of tumor immunity. VEGF could also impact the endothelial cells expression of immunological molecules. It decreases the expression of VCAM-1, which is important for the antitumor T cells adhesion and infiltration into tumors. Besides, VEGF also increases the expression of FAS ligand on endothelial cells, triggering apoptosis of T cells. VEGF also promotes the expansion of immune suppressive MDSCs, which further promotes the recruitment of Tregs. **d** EZH2 inhibition increases the production of CXCL9 and CXCL10, which are attractant cytokines promoting trafficking of T cells to tumor. Besides, EZH2 inhibition could selectively target intratumeral Tregs and reduce its immunosuppressive capacity. **e** In tumor cells, the ablation of LSD1 in cancer cells increases repetitive element expression, including ERVs, and decreases expression of RISC components. This leads to dsRNA stress and activation of type 1 interferon, which stimulates antitumor T-cell infiltration. In addition, inhibiting LSD1 in CD8^+^ T cells unleashes the transcription program mediated by TCF1, which is critical for the maintenance of the progenitor subset of intratumoral CD8^+^ T cells for persistent tumor control. dsRNA double-stranded RNA, ERVs endogenous retroviral elements, MDSCs myeloid-derived suppressor cells, RISC RNA-induced silencing complex, TCF1 T-cell factor 1, VCAM-1 vascular cell adhesion molecule-1

The hyperactive PI3K signaling, whether it is the consequence of PI3KCA mutations or PTEN deletions, can promote the establishment of tumor suppression by developing tumors,^467^ suggesting the potential use of PI3K inhibitor to enhance the efficacy of immunotherapy in the clinic (Fig. 6a). In preclinical models of melanoma, loss of PTEN in tumor cells inhibits T-cell-mediated tumor killing and restricts T-cell trafficking into tumors.^468^ A number of immunosuppressive cytokines, including CCL12 and VEGF are elevated in melanoma patients harboring *PTEN* loss. In this study, the lipidation of autophagosome protein LC3 and autophagy in tumor cells, which can decrease T-cell priming and modulate resistance to T-cell-mediated apoptosis, is also inhibited due to the loss of PTEN protein and activation of PI3K.^468^ Consistent with these findings, previous studies have also found that inactivation of PI3Kδ could break Tregs-mediated tumor immune tolerance, resulting in the activation of CD8^+^ T-cell responses and subsequent tumor regression.^469–472^ The Tregs T-cell receptor (TCR) downstream signaling, proliferation, and survival are dominantly dependent on PI3Kδ, but not PI3Kα or PI3Kβ.^470^ This potential adjuvant role for PI3Kδ inhibition in cancer immunotherapy was confirmed in a neoadjuvant, phase II clinical trial treatment-naive patients with resectable HNSCC.^117^ The inhibition of PI3Kδ by AMG319 decreased the number of tumor-infiltrating Tregs and activated intratumoral CD4^+^ and CD8^+^ T cells. However, the unfavorable safety profile should also be noticed, with frequent and severe grade 3/4 irAEs, probably driven by the systemic effect on Tregs in non-malignant tissues.^117^ Besides, as PI3K signaling is also essential in maintaining effector T-cell function,^473–475^ a systemic inhibition of PI3Kδ impairs the function of CD8^+^ cytotoxic T lymphocytes, which antagonizes ICB therapy intending to boost the CD8^+^ T-cell response, counteracting any advantages brought by impairing intratumoral Tregs.^476^ The protocols of administrating PI3Kδ inhibitors were considered as an essential part. A modified treatment regimen with intermittent dosing of PI3Kδ inhibitors has shown a comparable antitumor efficacy while limiting toxicity.^115–117^ In addition, given that PI3Kδ signaling might be required for signaling reactivation in exhausted T cells by ICB therapy,^477,478^ sequential combination treatment might be more effective. The study of Isoyama et al.^472^ confirmed that the combination protocol with anti-PD-1 mAb administrated first, followed by anti-PD-1 mAb plus PI3Kδ inhibitor induced the most effective and durable antitumor activity.

### ICB and CDK4/6 inhibition

CDK4/6 is regarded as a promising target in treating LSCC when appropriate candidate patients are identified by the downstream functional assessment.^38^ Recent studies have found that inhibition of CDK4/6 not only induces tumor cell-cycle arrest, but also increases T-cell inflammatory signature in tumors, which may act synergistically with ICB therapies^479,480^ (Fig. 6b). In mouse models, combining CDK4/6 inhibitors with PD-1 axis inhibitors resulted in significantly improved antitumor efficacy compared with either treatment alone.^480–483^ The mechanisms of this synergistic effects have been under extensive studies recently. Goel et al.^111^ found that the inhibition of CDK4/6 could increase the functional capacity of tumor cells to present antigens. Besides, CDK4/6 inhibitors could markedly and selectively reduce the immunosuppressive Tregs population.^111^ This preference of CDK4/6 inhibitor may be attributed to the higher expression level of RB by Tregs (3.1-fold higher in Tregs than in CD8^+^ T cells,^484^) a key modulator of CDK4/6 pathway.^485^ The inhibition of CDK4/6 in tumor cells also increases PD-L1 protein levels,^481^ which could be one of the mechanisms leading to the resistance of CDK4/6 inhibitor via evasion of immune checkpoints surveillance. This provides the rationale for the combination of PD-L1 blockade treatment and CDK4/6 inhibitors as a more potent antitumor treatment option. Properly timed and sequenced doses of CDK4/6 inhibitors could enhance T-cell activation^483^ and induce T-cell memory for maintaining long-term antitumor immunity.^486,487^ Despite the great efficacy shown in preclinical models, some clinical trials co-administering CDK4/6 inhibitors and ICB therapy were halted because of severe toxicity (e.g., NCT02779751,^488,489^ NCT04118036, NCT04075604). A different administrating method of CDK4/6 inhibitors has been proposed which may mitigate the toxicity risk.^486,487^ The capacity of CDK4/6 inhibitors to promote T-cell memory gives rationales for using CDK4/6 inhibitors as a preconditioning tool, priming the T-cell pool before the application of ICB. In mouse models, preconditioning tumor-bearing mice with a CDK4/6 inhibitor significantly improved the efficacy of anti-PD-1 ICB therapy.^486^ Taken together, these results suggest CDK4/6 inhibition in combination with immunotherapy is a promising therapeutic strategy but still needs further investigation.

### ICB and VEGF-VEGFR signaling blockade

With over 30 years of extensive research on VEGF, the biological role of VEGF has extended beyond its impact on neovascularization and angiogenesis, which also functions as an immunomodulator (Fig. 6c). It has been shown that multiple immune cells could be influenced by VEGF, including DCs, T cells, Tregs, and MDSCs.^490^ Both in animal models and humans, the inhibition of VEGF could increase the number of tumor-infiltrating lymphocytes.^491,492^ VEGF is also important for the expansion of the immunosuppressive MDSCs via its binding to VEGFR1, which further promotes an immunosuppressive microenvironment by de novo development of Tregs.^493^ In renal cell carcinoma, the inhibition of VEGF-VEGFR signaling by either mAbs or VEGF-TKIs could reverse MDSC-mediated immunosuppression.^494,495^ This immunosuppressive profile of the VEGF/VEGFR axis is also confirmed by the fact that VEGF-A-VEGFR pathway blockade inhibits tumor-induced proliferation of Tregs.^496,497^ Aberrant angiogenesis within the TME can mediate immune escape and reduce the efficacy of immunotherapy by hampering the delivery of drugs, oxygen, and effector T cells.^498,499^ This tumor hypoxia promotes the recruitment of Tregs, which facilitates angiogenesis through excess production of VEGF.^500^ In hand with other tolerogenic leukocyte populations such as MDSCs^501^ and pDCs^502^ which also produce VEGF and support angiogenesis, this leads to further tumor tolerance and growth. During adaption to the hypoxic TME, tumor-infiltrating cytotoxic T cells are characterized by high glycolytic rates.^503,504^ A recent study has found that hypoxia-inducible transcription factor 1α (HIF1α) is essential for the effector state in CD8^+^ T cells.^505^ VEGF-deficient CD8^+^ T cells showed lower efficiency on infiltrating tumors. An HIF1α/VEGF axis has been proposed in cytotoxic T cells to regulate tumor progression.^505^ The above-mentioned findings provide further evidence for the ongoing clinical evaluation of combined immunotherapies and antiangiogenic approaches. Currently, the only approved combination therapy of immunotherapy and antiangiogenic approach for NSCLC is the PD-L1 inhibitor atezolizumab in combination with bevacizumab, paclitaxel, and carboplatin for the first-line treatment of patients with metastatic non-squamous NSCLC with no EGFR or ALK genomic tumor aberrations.^506^ AK112, a tetrameric bispecific antibody targeting PD-1 and VEGF, has shown in a phase I dose-escalation study with a manageable safety profile.^507^ It is currently being evaluated in a phase II clinical trial for stage IIIB/C or IV NSCLC (NCT04736823).

### ICB and epigenetic therapies

Recent studies revealed the prominent role for modulating immune cells and regulating anticancer immune response by epigenetic therapy, which nominates these epidrugs a new category of immune modulators.^508–513^ These preclinical findings have led to the evaluation of various epidrugs and immunotherapy combinations in clinical trials for treatment of LSCC (Table 2). Some combinations of epidrugs and ICB therapy in early-stage clinical trials have demonstrated significant clinical activity and acceptable safety.^514^ In this part, we will discuss the combination of ICB therapy with inhibition of potentially effective epigenetic targets in LSCC.

An important epigenetic modulator in LSCC is the H3K27 methyltransferase EZH2, whose dependency was observed in *SOX2* amplified LSCC cell lines.^38^ Like other epigenetic modulators, EZH2 also plays an important role in mediating the immune response to the tumor both in tumor cells and immune cells (Fig. 6d). Previous studies have found that EZH2 can repress the expression and subsequent production of Th1-type chemokines CXCL9 and CXCL10, thus preventing efficient T-cell trafficking to tumors, a process that can be reversed by EZH2 inhibition in combination with anti-PD-L1 checkpoint blockade.^513,515^ Through its epigenetic reprogramming of T-cell antigen-presenting gene, EZH2 has been shown to be a driver of resistance to cancer immunotherapy.^516–519^ Besides, EZH2 plays an important role in maintaining the identity of immunosuppressive Tregs during cellular activation, where it is recruited by Foxp3 to repress key genes.^520,521^ Disruption of EZH2 activity in Tregs can reprogram the tumor-infiltrating Tregs for pro-inflammatory activities, thereby enhancing the recruitment and function of CD8^+^ and CD4^+^ effector T cells to eliminate tumors.^522^ The dual functions of EZH2 in repressing antigen presentation and altering Tregs functions makes the EZH2 inhibition a rational strategy in combination with CTLA-4 inhibitors or PD-1 axis inhibitors, respectively. Indeed, the additional efficacy of both combination strategies was confirmed in preclinical mouse models.^523–525^ This synergistic effect of EZH2 blockade and ICB therapy is currently being validated in multiple clinical trials (e.g., NCT02220842,^526^ NCT03854474,^527^ NCT04407741).

As a direct regulator of *SOX2*, which is a lineage-survival oncogene of LSCC,^40^ inhibition of LSD1 is regarded as a promising treatment strategy for LSCC.^38^ Recently, studies have also found that this epigenetic regulator is important in regulating tumor immunity (Fig. 6e). In 2018, Sheng et al.^510^ found that the inhibition of this histone demethylase in cancer cells resulted in double-stranded RNA (dsRNA) stress and activation of type 1 interferon, thereby stimulating potent antitumor T-cell immunity. Furthermore, the inhibition of LSD1 could elicit significant response to anti-PD-1 therapy in ICB-refractory mouse melanoma.^510^ Consistently, TCGA data analysis reveals an inverse correlation between LSD1 expression and CD8^+^ T-cell infiltration in various human cancers.^510^ In the same year, Qin et al.^528^ also found that combining LSD1 inhibitors with anti-PD-1 mAbs significantly suppressed tumor growth and pulmonary metastasis in mice bearing TNBC xenograft tumors, whereas anti-PD-1 mAbs alone failed to elicit an obvious anticancer effect. Over the next few years, the enhancement of immunotherapy efficacy through inhibiting LSD1 was also demonstrated in several other tumors.^529–531^ The mechanisms of LSD1 ablation in tumor immunity have not been clearly elucidated. According to Liu et al.,^532^ the inhibition of LSD1 in T cells increases the persistence of the progenitor-exhausted CD8^+^ T cells through augmenting the transcriptional network controlled by T-cell factor 1 (TCF1), which is essential for maintaining the progenitor phenotype. These progenitor-exhausted CD8^+^ T cells are characterized by high proliferation capacity, which gives rise to more differentiated cells with strong cytotoxicity. These properties make progenitor-exhausted CD8^+^ T cells a major determinant of responses to PD-1 axis blockade.^533–536^ Based on this synergistic effect of LSD1 inhibitors and PD-1 axis inhibitors, there is already an ongoing clinical trial evaluating this combination therapy in SCLC and LSCC patients (NCT04350463). Another study has proposed the addition of blocking TGF-β in this combination therapy.^537^ The efficacy of this triple combination therapy has been validated in certain poorly immunogenic or “cold” tumors.^537^ However, the safety and efficacy of this combination strategy still need to be verified in future studies.

## Conclusions and future perspectives

Accounting for ~30% of all NSCLC, LSCC remains a leading cause of death with few therapeutic options.^3,4^ While targeted therapies demonstrated significant benefits in LUAD patients, patients with LSCC have not benefited from targeted therapy due to the distinct nature of LSCC.^20,538^ The advent of immunotherapies has significantly improved the prognosis of patients with LSCC, and this burgeoning field of cancer immunotherapy continues to grow as new druggable targets are discovered. With comprehensive proteogenomic data, the established LSCC biology can be more deeply elucidated, potentially uncovering new potential implicated therapeutics targets.^38^

In this review, we discussed some new insights in some signaling pathways which have been proved in other cancer types, like PI3K pathway, CDK4/6 pathway and VEGF/VEGFR signaling. The two key squamous differentiation markers *SOX2* and *TP63* offer the chance of therapeutic targets in LSCC. SOX2 was considered undruggable before, thus intensifying therapeutic interests in upstream or downstream targets, including LSD1 and EZH2. Newly identified epigenetic targets, like NSD3, were also emerging as potential targets in treating LSCC. Having shown great benefits in LSCC, ICB therapies still faced the problems of a relatively low response rate and high rate of irAEs in some cases. We discussed some newly discovered mechanisms of these immune checkpoints which may be useful in tackling these problems. Combinations of different ICB therapies or ICB therapy and other targeted therapies have emerged as an appealing treatment paradigm. Whether it is the combination of ICB therapies plus epigenetic therapies, or ICB therapies plus VEGF-VEGFR inhibitor, it represents an innate inner connection among these different signaling pathways. With the persistent exploration of these complex biological interactions among different signaling pathways, it will surely provide exciting opportunities for new, improved and personalized therapeutic interventions in LSCC patients. Multi-omics clustering has identified five LSCC molecular subtypes^38^ and this heterogeneity of LSCC reveals the fact that combination therapies targeting more than one target or signaling pathway may yield more therapeutic choices. In the last decade, ICB therapies have made a major breakthrough in improving the prognosis of LSCC patients both in the first- and second-line settings. We believe immunotherapy will remain the pillar of LSCC treatment. Meanwhile, the clinical translation of other novel therapeutic targets is still in a great demand which may improve the efficacy of current ICB therapy-based regimens.
